# A short exposure to a semi-natural habitat alleviates the honey bee hive microbial imbalance caused by agricultural stress

**DOI:** 10.1038/s41598-022-23287-6

**Published:** 2022-11-06

**Authors:** June Gorrochategui-Ortega, Marta Muñoz-Colmenero, Marin Kovačić, Janja Filipi, Zlatko Puškadija, Nikola Kezić, Melanie Parejo, Ralph Büchler, Andone Estonba, Iratxe Zarraonaindia

**Affiliations:** 1 Department of Genetics, Physical Anthropology and Animal Physiology, University of the Basque Country (UPV/EHU), Barrio Sarriena s/n, 48940 Leioa, Spain; 2 Instituto de Investigaciones Marinas (CSIC)/Institute of Marine Research, Eduardo Cabello 6, 36208 Vigo, Pontevedra Spain; 3 Faculty of Agrobiotechnical Sciences Osijek, Josip Juraj Strossmayer University of Osijek, V.Preloga 1, 31000 Osijek, Croatia; 4 Department of Ecology, Agronomy and Aquaculture, University of Zadar, Trg Kneza Višeslava 9, 23000 Zadar, Croatia; 5 Department of Fisheries, Apiculture and Special Zoology, Faculty of Agriculture, University of Zagreb, Svetošimunska Cesta 25, 10000 Zagreb, Croatia; 6 Landesbetrieb Landwirtschaft Hessen (LLH), Bieneninstitut, Erlenstraße 9, 35274 Kirchhain, Germany; 7 IKERBASQUE, Basque Foundation for Science, Bilbao, Spain

**Keywords:** Microbial ecology, Bacteria, Microbial communities

## Abstract

Honeybee health and the species’ gut microbiota are interconnected. Also noteworthy are the multiple niches present within hives, each with distinct microbiotas and all coexisting, which we termed “apibiome”. External stressors (e.g. anthropization) can compromise microbial balance and bee resilience. We hypothesised that (1) the bacterial communities of hives located in areas with different degrees of anthropization differ in composition, and (2) due to interactions between the multiple microbiomes within the apibiome, changes in the community of a niche would impact the bacteria present in other hive sections. We characterised the bacterial consortia of different niches (bee gut, bee bread, hive entrance and internal hive air) of 43 hives from 3 different environments (agricultural, semi-natural and natural) through 16S rRNA amplicon sequencing. Agricultural samples presented lower community evenness, depletion of beneficial bacteria, and increased recruitment of stress related pathways (predicted via PICRUSt2). The taxonomic and functional composition of gut and hive entrance followed an environmental gradient. *Arsenophonus* emerged as a possible indicator of anthropization, gradually decreasing in abundance from agriculture to the natural environment in multiple niches. Importantly, after 16 days of exposure to a semi-natural landscape hives showed intermediate profiles, suggesting alleviation of microbial dysbiosis through reduction of anthropization.

## Introduction

The Western honey bee (*Apis mellifera*) is at risk^1,2^ due to multiple stressors, including but not limited to poor nutrition, pesticides, and pathogens^3^. Though not unique to agricultural areas, these stressors are typical of anthropized lands (i.e. areas under human influence), where most beekeeping practices are performed. Thus, anthropized areas are an ideal place to study honey bee microbiota dysbiosis and its recovery. The microbial realm associated with honey bees and their beehives is essential to maintain host and community homeostasis^4,5^, and the host immune response is intrinsically associated with native gut microbiota^4,6^. Symbiotic interactions between gut microbiota and bees can be highly specialised, whereby some bacteria and fungi prevent pathogen colonisation, clear toxic metabolites, or metabolise substances otherwise unavailable to bees^4,6–12^. The core gut microbiota of honey bee workers is composed of 8–10 bacterial species^13^ and can be disrupted by external microbes, nutrient shortage or chemicals^14–16^, with the latter harming honey bees at all stages of development^17–20^. Dysbiosis of the intestinal flora can compromise honey bee resilience and cause disease onset, while positive reinforcement induces recovery of beneficial microbial profiles and strengthens host health^6^. Recuperation of the core microbiota could be achieved through probiotic supplementation of core gut bacteria^21^. However, there is no consensus on what causes gut microbiota dysbiosis, its regulation and recovery, and how it affects (and is affected by) other hive microbial communities.

Within the hive, multiple microenvironments or niches (e.g. food reserves), each with their unique microbiotas, coexist alongside bees and their gut. These niche microbiotas are non-isolated and can undergo alterations when encountering other microbial communities or stressors. Habitat and bee diet can also affect some of these niche communities, as seen in corbicular bee pollen^22^ and in bee bread, where the microbial community composition changes from initial environmental profiles towards bee bread-specific communities^23^. It is equally important to consider the effect of stressors, habitat and diet, in other niches such as airborne particles or hive surfaces. Pesticides not only directly affect honey bees but can also accumulate to hazardous concentrations within hive components^20,24^, disrupting the microbiota of these niches and weakening colonies^18^. Social interactions among colony integrants could also facilitate distribution of microbes within hives^25^, as seen with the *Arsenophonus* genus^26^, or enable intercolony pathogen transmission^27,28^. Due to the close relationship between bees, the colony and hive components, stressor-mediated dysbiosis of hive niche microbiotas could be transmitted to the honey bee gut microbiota. Indeed, Anderson et al.^29^ found gut-specific bacteria in hive samples, which suggested bacterial transmission between the honey bee gastrointestinal tract and hive internal microenvironments. We propose the concurrent study of multiple niche-specific microbiotas, to better understand their role in this beehive-bee interconnectedness. We nominate the term "apibiome" to refer to the microbial community formed by all beehive niches, including the bees.

Herein, we characterised the in-field bacterial consortia and the predictive functional profile of the bacterial fraction of the apibiome, particularly of the honey bee gut, bee bread, hive entrance and internal hive air, of beehives in contrasting environments, through V4 16S rRNA Illumina amplicon sequencing. Functional characterization was performed using predictive PICRUSt2. Based on the results obtained by Muñoz-Colmenero et al.^30^, wherein contrasting anthropic habitats were compared, we expected higher anthropization levels to increase opportunistic/pathogenic bacterial loads and deplete beneficial bacteria, whilst lower anthropization would result in more balanced gut microbiotas. The contrasting environments studied here consisted of an agricultural, a semi-natural and a natural area. Agricultural and semi-natural areas contained newly formed colonies of the same origin, while colonies located in the natural area had survived over 10 years without human interference and have remained tolerant to the presence of *Varroa destructor* infections. To the best of our knowledge, this is the first study testing the effect of anthropization in multiple beehive microbiomes (i.e. the apibiome).

## Results

### Measurement of colony strength traits and *Varroa destructor* load

**Figure 1:**
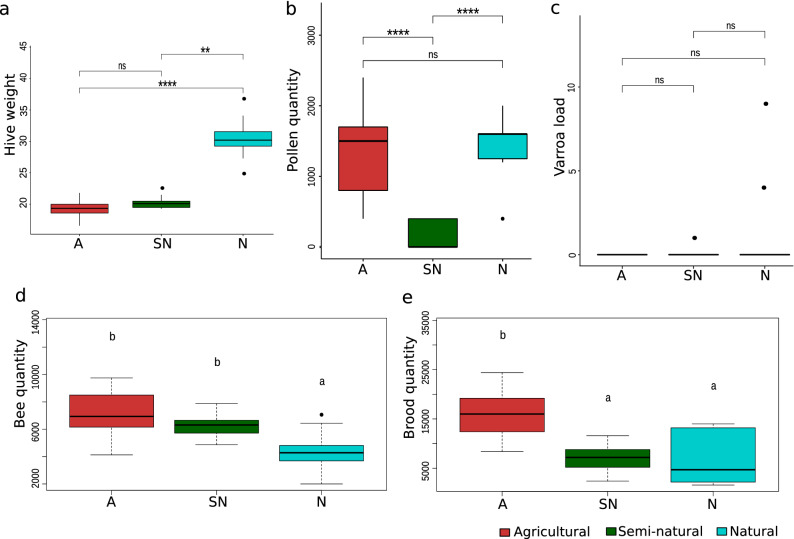
Statistical comparison between environments for colony strength traits and *Varroa destructor* loads. The top plots (**a**–**c**) show pairwise Dunn tests with Benjamini–Hochberg correction, with significance expressed as 0 (****), 0.001 (***), 0.01 (**), 0.05 (*), no-significant (ns). The bottom plots (**d**,**e**) show pairwise post-hoc Tukey’s test, with different letters indicating statistically significant differences. (**a**) Hive weight measured in kilograms (Kruskal–Wallis test, *χ2(2)* = 26.58, *p* < 0.0001). (**b**) Estimated pollen cells per apiary (Kruskal–Wallis test, *χ2(2)* = 24.08, *p* < 0.0001). (**c**) *Varroa destructor* load measured by the Powdered Sugar method (Kruskal–Wallis test, *χ2(2)* = 4.72, *p* > 0.05). (**d**) Bee population measured as the number of bees per colony (ANOVA, *F* = 13.64, *p* < 0.0001; pairwise Tukey, for A vs N *p* < 0.001, for SN vs N *p* < 0.05). (**e**) Brood population measured by quantification of brood cells (ANOVA, *F* = 23.35, *p* < 0.0001; pairwise Tukey, for A vs SN *p* < 0.0001, for A vs N *p* < 0.0001).

Hives located in the natural area (henceforth natural hives or colonies) weighed the most (hive weight, Kruskal–Wallis test, *p* < 0.0001) (Fig. 1a) yet had the smallest bee population (ANOVA test, *p* < 0.0001) (Fig. 1d), excluding one colony with higher bee loads (i.e. outlier). Pollen was nearly absent in hives situated in the semi-natural area (i.e. semi-natural hives or colonies) compared with the pollen-rich agricultural (henceforth agricultural hives or colonies) and natural colonies (pairwise Dunn test, *p* < 0.0001) (Fig. 1b). Brood presence was highest in agriculture (ANOVA test, *p* < 0.0001) and very similar for natural and semi-natural ambients (pairwise Tukey test, *p* > 0.05) (Fig. 1e). Mean *Varroa* load was null and equal in all apiaries (Kruskal–Wallis test, *p* > 0.05) but two natural colonies had high mite loads (Fig. 1c).

### Comparison of bacterial community richness and taxonomic composition between hive niches

In total, 158 samples were adequately amplified and sequenced. After pair-end sequence assembly, quality filtering and singletons removal, our final dataset consisted of 7,962,468 reads. The total frequency of reads was 4,040,931 for gut (mean = 93,975.139 ± 29,760), 1,089,501 for bee bread (mean = 26,573.195 ± 13,298), 2,471,615 for hive entrance (mean = 58,847.976 ± 19,523), and 360,421 for internal air (mean = 11,263.156 ± 9,867).

Hive niche had a significant impact on bacterial community biodiversity (Faith Phylogenetic Diversity, PD), Shannon’s diversity index (H) and Pielou’s evenness index (J′) (Kruskal–Wallis *p* < 0.0001). Gut samples presented the least diverse (lowest PD and H, *p* < 0.0001) but most evenly distributed bacterial microbiome of all sample types (highest J′ *p* < 0.05) (Supplementary Fig. S1).

**Figure 2:**
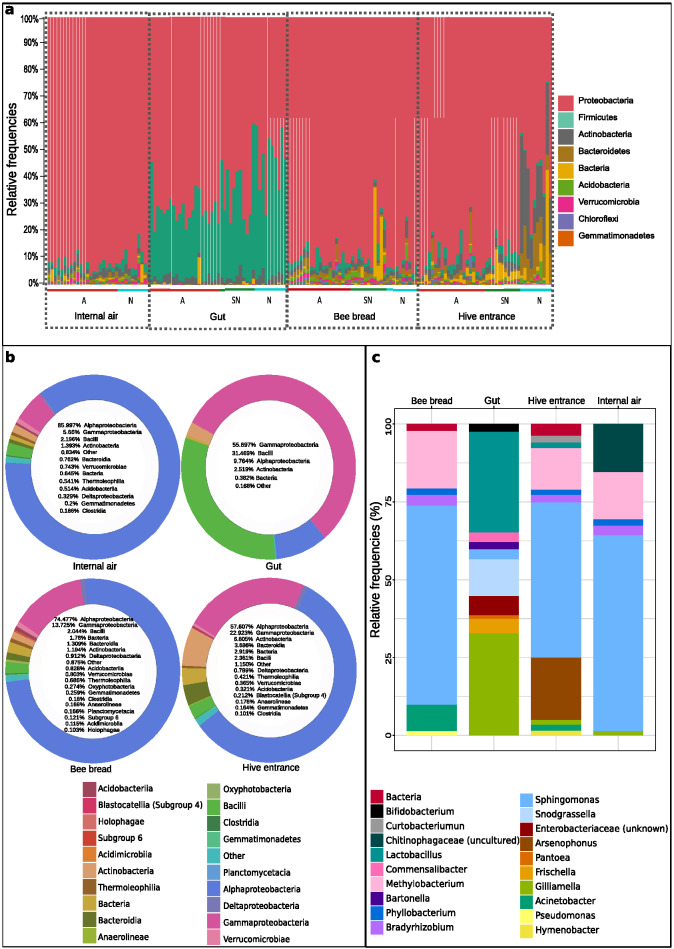
Bacterial communities of the studied hive niches at class and phylum levels. (**a**) Bar plots of the relative frequencies of the bacterial phyla present in each hive niche. Proteobacteria were the most abundant, excluding some natural and semi-natural gut samples rich in Firmicutes, as well as several natural hive entrances enriched for Actinobacteria and Bacteroidetes (A = agricultural apiary, SN = semi-natural apiary, N = natural apiary). (**b**) Pie charts of the bacterial classes presenting relative abundances ≥ 0.1%, in percentages. Some ASVs were only classified up to Domain level, and grouped as “Bacteria”. The group “Others” includes all additional taxa. Gut samples displayed a distinct microbial profile. Internal air, bee bread and hive entrance had similar abundances, with enrichment of Alphaproteobacteria, Actinobacteria and Bacteroidia; and numerous bacteria less than 0.1% abundant. (**c**) Most abundant bacterial genera (≥ 1.0%) per sample type, in percentages. Internal air was not sampled in the semi-natural apiary due to methodological constraints.

Proteobacteria were the most abundant taxa (Fig. 2a), being Alphaproteobacteria the most abundant class (in all niches except for gut), followed by Gammaproteobacteria, Bacilli and Actinobacteria (Fig. 2b). Internal hive air samples were overrun by Alphaproteobacteria [86%, represented by genera *Sphingomonas *(54.9%), *Methylobacterium *(13.2%)*, Bradyrhizobium* (2.66%), and *Phyllobacterium* (1.82%)] (Fig. 2c, Supplementary Fig. S2), and only Gammaproteobacteria (5.66%), Bacilli (2.2%) and Actinobacteria (1.4%) classes had relative abundances over 1% (Fig. 2b). Bee bread microbiotas were rich in Acidobacteria (0.83%), Verrucomicrobiae (0.80%), Thermoleophilia (0.69%) and Oxynphotobacteria (0.27%) families (Fig. 2b), and *Methylobacterium* (15.06%), *Acinetobacter* (6.79%) and *Pseudomonas* (1.06%), and shared similar abundances of *Sphingomonas*, *Bradyrhizobium* and *Phyllobacterium* with air samples (Fig. 2c, Supplementary Fig. S2). Hive entrance presented the highest relative abundances of the Actinobacteria (6.81%), Bacteroidia (3.69%) and Blastocatellia (0.21%) families among all hive niches, as well as unknown bacteria (2.92%) (Fig. 2b) and the *Arsenophonus* (15.64%), *Curtobacterium* (1.81%) and *Hymenobacter* (1.21%) genera (Fig. 2c, Supplementary Fig. S2). The bacterial microbiome composition of gut samples was skewed towards Gammaproteobacteria (55.7%) class and the Firmicutes phylum (31.47% Bacilli) presence, and was rich in classes with < 0.1% relative abundance (Fig. 2b). At genus level, over 70% of the gut microbiome was formed by the genera *Gilliamella*, *Lactobacillus* and *Snodgrasella* (Fig. 2c, Supplementary Fig. S2).

### Environmental and anthropic effects on diversity in different hive niches

Microbial communities were in general more evenly composed in the natural environment (Pielou’s index, Kruskal–Wallis test, *p* < 0.05 for gut, *p* < 0.01 for hive entrance), with semi-natural samples showing intermediate values (Supplementary Fig. S3a). Phylogenetic diversity changed for hive entrance samples, with natural hives having the highest values and anthropic the lowest (Kruskal–Wallis *p* < 0.05) (Supplementary Fig. S3b). There were also significant differences in Shannon’s diversity when comparing hive entrances located in agricultural versus natural landscapes (Kruskal–Wallis *p* < 0.01) (Supplementary Fig. S3c).

**Figure 3:**
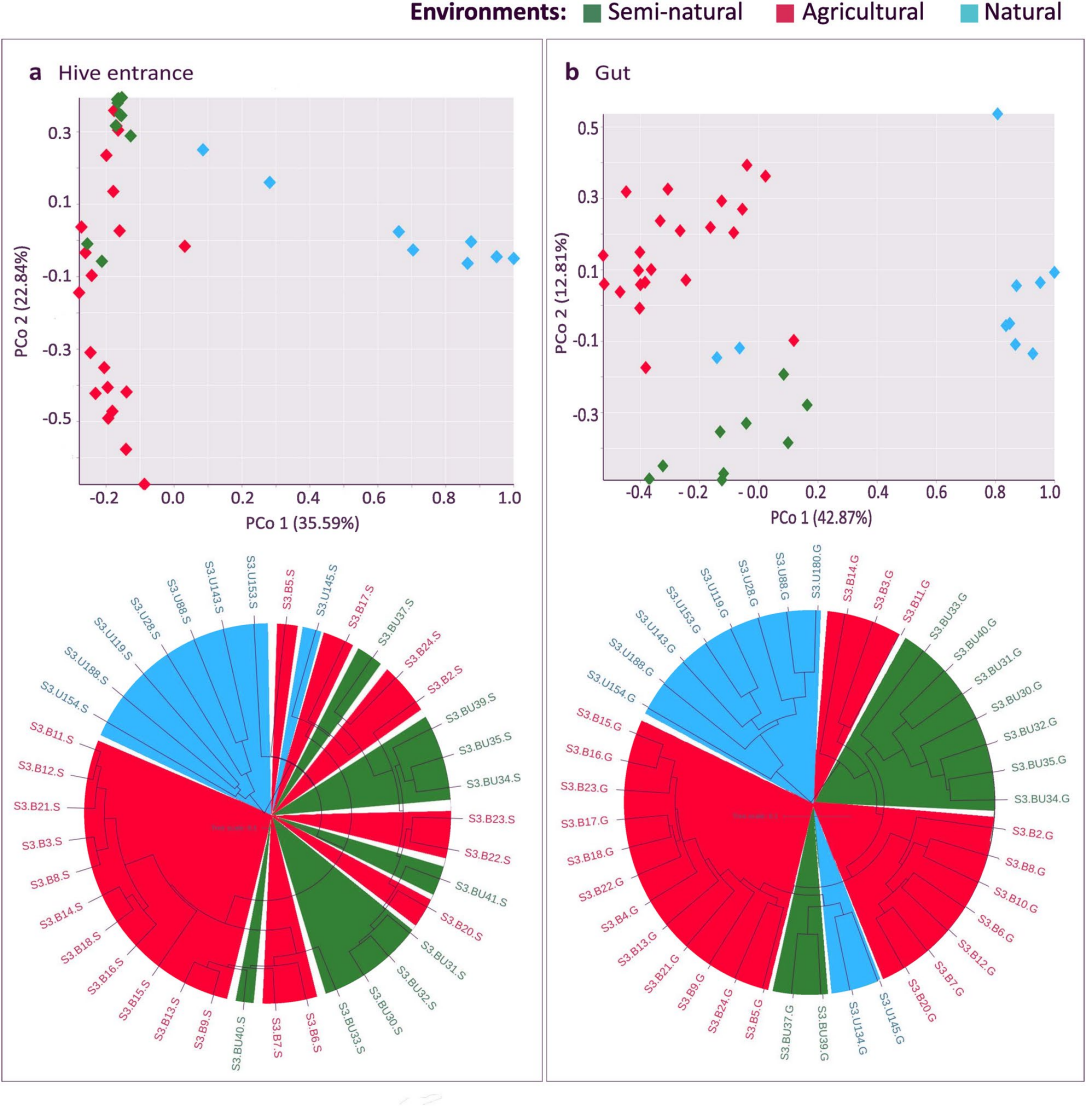
Beta diversity analysis of bacterial microbiota for hive entrance and gut samples. (**a**) Bray–Curtis based Principal Coordinate Analysis (PCoA) and UPGMA tree of hive entrance samples. Natural hives form an isolated cluster in the PCo1 axis. (**b**) Bray–Curtis based Principal Coordinate Analysis (PCoA) and UPGMA tree of adult worker gut samples, with clustering and isolation of all environments, and special differentiation for natural samples. Plotting: PCoAs were plotted using Vega editor (v5.22.1, https://vega.github.io/editor/#/). UPGMA trees were visualized in iTOL (v6.5.8, https://itol.embl.de/) and internal colors added via INKSCAPE (v0.92.3-1, https://inkscape.org/).

Bacterial community composition significantly differed by environment for bee bread, hive entrance and gut samples (PERMANOVA, p ≤ 0.01 for all, pseudo-F > 4, 11, 14 respectively) but not for internal air (Supplementary Table S1). Bee bread community differences between environments were significant but weak, with a slight clustering in the PCoA (Supplementary Fig. S4a) and a lack of clustering in the UPGMA (Supplementary Fig. S4b). On the contrary, hive entrance samples showed a differentiated cluster formed by natural samples and a significant division between anthropic and semi-natural environments (pairwise PERMANOVA, *pseudo-F* = 7.63, *p* = 0.002) (Supplementary Table S1) reflected in clustering of environments on UPGMA, but not clearly visible in the PCoA (Fig. 3a). However, heterogeneous dispersion by environment was found to be significant (betadisper, *F* = 9.941, *p* = 0.001) with the natural group showing intra compositional variance. Worker guts also clustered separately for natural samples and were the only sample type showing a clear microbial clade divergence between agricultural and semi-natural environments (PCo2 = 12.81% and PERMANOVA *pseudo-F* = 8.86, *p* = 0.001) (Fig. 3b) (Supplementary Table S1). However, the largest Bray–Curtis distance (an index that measures between samples microbial compositional dissimilarity) was found when comparing either anthropic or semi-natural against natural samples (PCo1 = 42.87% and PERMANOVA *pseudo-F* > 10, *p* = 0.001) (Supplementary Table S1).

### Functional and bacterial community profiles across environments

#### Honey bee worker gut samples

**Figure 4:**
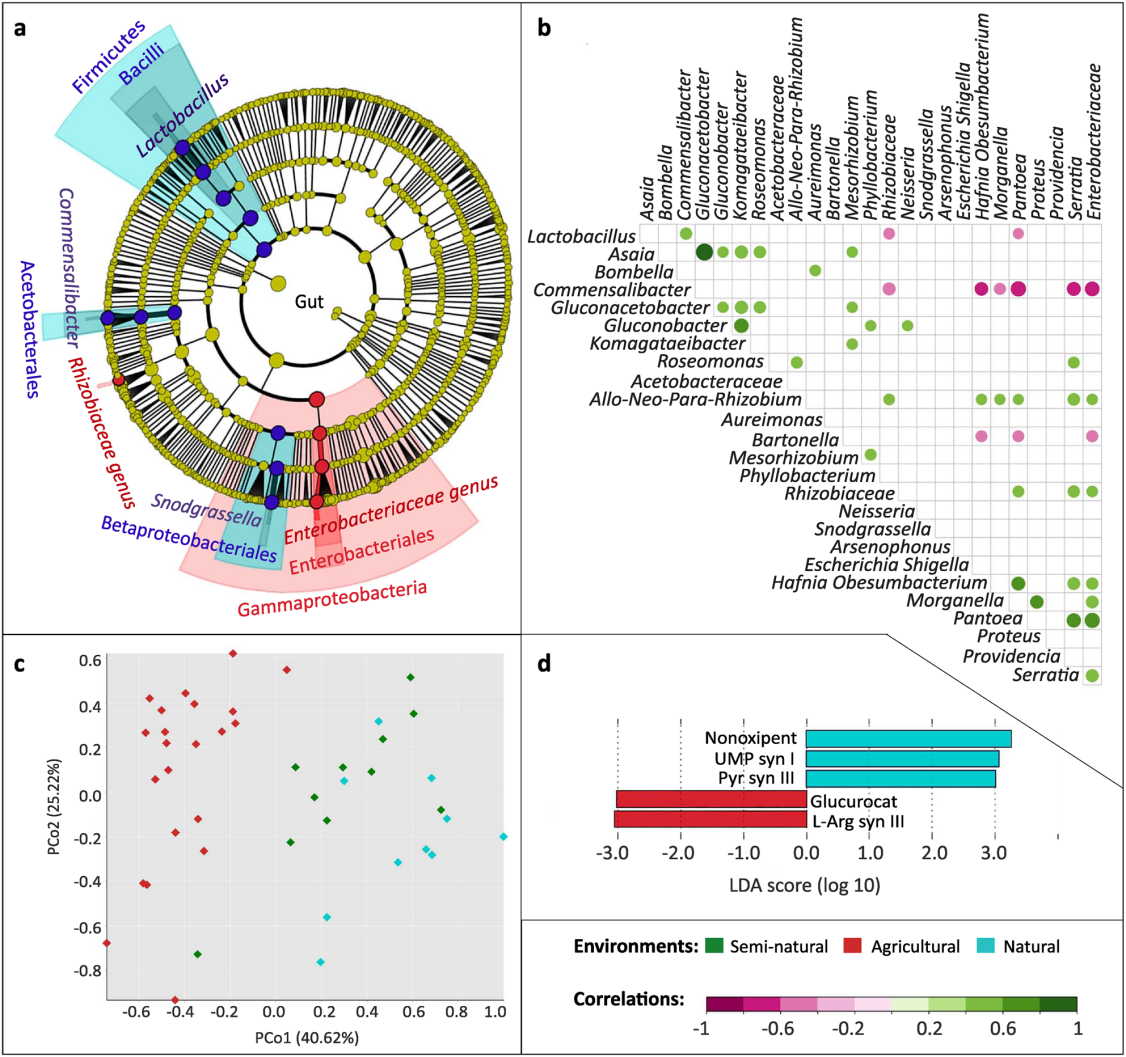
Characterization of bacterial communities of worker gut samples. (**a**) Cladogram of significantly enriched bacteria in each environment, from phylum to genus level, according to LEfSe. LEfSe cladogram results are plotted according to phylogeny. The outermost circle corresponds to the lowest taxonomic rank (genera, level to which ASVs were collapsed). From there, each circle equals a higher taxonomic rank, with phyla being the inner circle. Only significant taxa are plotted. (**b**) Spearman correlation analysis of the honey bee gut bacterial communities at *p* < 0.05. (**c**) Principal Coordinate Analysis (PCoA) of samples according to the predictive functional profile. (**d**) Significantly expressed MetaCyc pathways (predicted functional profile) according to LEfSe. The bigger the LDA value obtained for a feature, the more significant. Only significant features are plotted in the histogram. (**d**) Nonoxipent: Pentose phosphate pathway I (non-oxidative branch), UMP syn I: UMP biosynthesis I, Pyr syn III: Pyrimidine deoxyribonucleotides de novo biosynthesis III, Glucurocat: β-d-glucuronosides degradation, L-Arg syn III: l-arginine biosynthesis III (via N-acetyl-l-citrulline). Plotting: Cladograms and histograms of LEfSe results were plotted in Galaxy (web application, https://huttenhower.sph.harvard.edu/galaxy/) and taxa names cleaned with INKSCAPE (v0.92.3-1, https://inkscape.org/). PCoAs were plotted using Vega editor (v5.22.1, https://vega.github.io/editor/#/).

All three environments had a strong representation of Firmicutes (*Lactobacillus*), Gammaproteobacteria (the Orbaceae *Gilliamella* and *Frischella*), Betaproteobacteria (*Snodgrasella*), Actinobacteria (*Bifidobacterium*) and Alphaproteobacteria (*Bartonella, Methylobacterium, Sphingomonas* and *Bradyrhizobium*). However, there were clear differences between natural and agricultural environments. Enrichment of the Enterobacteriaceae (Gammaproteobacteria) and Rhizobiaceae (Alphaproteobacteria) families was detected in the agricultural environment (*LDA* > 3.6) (Fig. 4a). The Gammaproteobacteria genera *Pantoea *and *Arsenophonus* were also enriched in the agricultural environment compared to the other environments, albeit not significantly (Supplementary Table S2). The genera *Lactobacillus* (Bacilli), *Commensalibacter* (Alphaproteobacteria) and *Snodgrasella* (Gammaproteobacteria) represented the natural environment (*LDA* > 4.6) (Fig. 4a). Interestingly, the *Gilliamella* genus was underrepresented in natural samples, with overall lower relative frequencies than in the other two environments (median 16.94% in natural, 34.28% in semi-natural and 37.16% in agricultural) (Supplementary Table S2). Semi-natural samples were characterised by a higher presence of *Bartonella* (sparse in the other environments) and *Frischella* (median 8.24% versus agricultural 2.91% and natural 3.54%) (Supplementary Table S2). No differently enriched taxa were found in the semi-natural habitat (Fig. 4a), but importantly most agricultural and natural representatives had intermediate abundances in this environment (Fig. 6a).

Concerning bacteria-bacteria interactions, the natural representatives *Commensalibacter* and *Lactobacillus* were positively correlated (*R* = 0.50, *p* < 0.01). Several genera from the Enterobacteriaceae family (*Hafnia-Obesumbacterium, Pantoea* and one non-assigned) possessed high negative correlations with *Commensalibacter* (*R* < − 0.62, *p* < 0.001) and more moderate correlations with *Bartonella* (*R* < − 0.46, *p* < 0.05), which was more abundant in semi-natural colonies. Other Enterobacteriaceae (*Morganella, Serratia*) presented moderate negative correlations with *Commensalibacter* alone (*R* < − 0.48, *p* < 0.05). Overall, Enterobacteriaceae bacteria promoted the presence of other Enterobacteriaceae, especially between bacteria negatively correlated with *Commensalibacter.* Two Rhizobiaceae were also positively correlated with several Enterobacteriaceae (*R* > 0.4, *p* < 0.05). High positive correlations were also observed for several Acetobacteraceae genera, with the highest correlations present among *Asaia* versus *Gluconacetobacter* (*R* = 1, *p* < 0.0001); and *Gluconobacter* versus *Komagataeibacter* (*R* = 0.70, *p* < 0.001) (Fig. 4b).

In the predictive functional profile, natural and agricultural environments presented either the lowest or highest recruitment of features. Semi-natural samples generally had intermediate values, indicating a possible “intermediate microbiome” found in semi-natural gut samples. Similarity analysis via PCoA demonstrated clustering of environments along the PCo1 axis (Fig. 4c). All environments had similar biodiversity values, with agricultural samples having the highest H values against both natural and semi-natural (*p* < 0.001) and no significant differences found between semi-natural and natural samples (Supplementary Fig. S5a). Natural samples indicated significant recruitment of several anabolic reactions for the generation of precursor metabolites, nucleosides and nucleotides, while Arginine biosynthesis and β-D-glucuronoside degradation pathways were more representative of agricultural samples (Fig. 4d). The semi-natural environment retained no significant pathways, even though it had intermediate abundances for every agricultural- and natural-significant pathway detected.

#### Hive entrance samples

**Figure 5:**
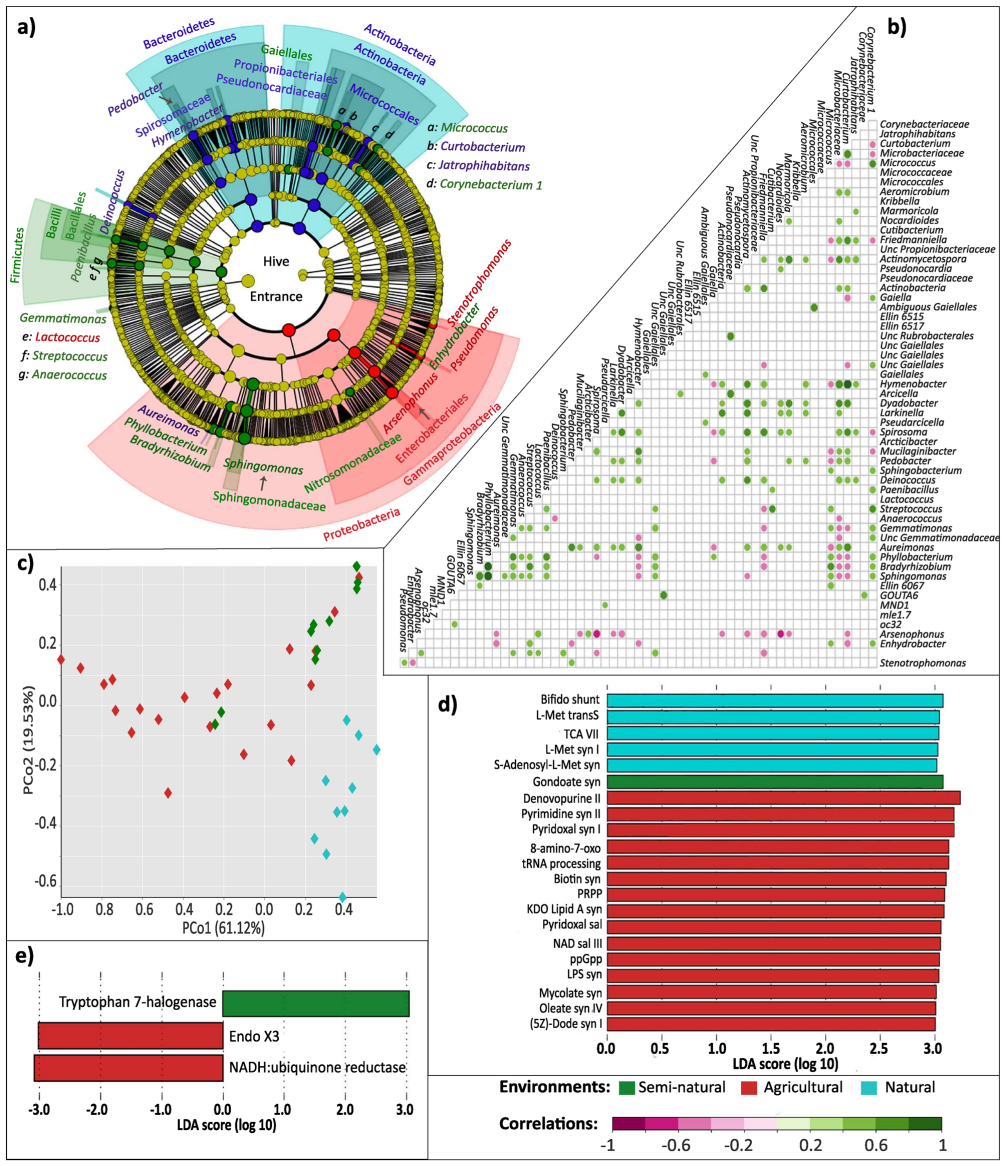
Characterization of the bacterial communities in hive entrance samples. (**a**) Significantly enriched bacteria in each environment, according to LEfSe. Agricultural hives were rich in Gammaproteobacteria and *Lactococcus*. The classes Actinobacteria and Bacteroidia were prevalent in natural samples. Semi-natural samples were enriched in the *Sphingomonas* genus (*LDA* > 5.0), the Bacilli class, genera from the Alphaproteobacteria (*Bradyrhizobium, Phyllobacterium*) and Gammaproteobacteria (*Enhydrobacter*) classes, as well as genera from the Firmicutes, Gemmatimonadetes (*Gemmatimonas* and an uncultured genus), and Actinobacteria phyla. (**b**) Spearman correlation analysis at *p* < 0.05. Positive values were particularly high for *Curtobacterium/Hymenobacter*, *Phyllobacterium/Sphingomonas* and *Phyllobacterium/Bradyrhizobium* interactions (*R* > 0.80, *p* < 0.0001). The most negative interactions were found between *Arsenophonus/Spirosoma* and *Arsenophonus/Nocardioides* (R ⋍ − 0.6, *p* < 0.001). (**c**) Principal Coordinate Analysis (PCoA) of samples according to the predictive functional profile (MetaCyc pathways). (**d**) Significantly recruited functions according to LEfSe. (**e**) Significantly enriched enzymes according to LEfSe. The enzymes Endo X3 (EC 3.1.22.4) and coenzyme Q reductase (EC 7.1.1.2, formerly EC 1.6.5.3) were agricultural representatives, while tryptophan 7-halogenase (EC 1.14.19.9) was enriched in semi-natural samples. Bifido shunt: Bifidobacterium shunt, L-Met transS: l-methionine biosynthesis (transsulfuration), TCA VII: TCA cycle VII (acetate-producers), L-Met syn I: l-methionine biosynthesis I, S-Adenosyl-l-Met: S-adenosyl-l-methionine biosynthesis, Gondoate syn: gondoate biosynthesis (anaerobic), Denovopurine II: purine nucleotides de novo biosynthesis II, Pyrimidine syn II: pyrimidine deoxyribonucleotides de novo biosynthesis II, Pyridoxal syn I: pyridoxal 5′-phosphate biosynthesis I, 8-amino-7-oxo: 8-amino-7-oxononanoate biosynthesis I, tRNA processing: tRNA processing, Biotin syn: biotin biosynthesis I, PRPP: histidine, purine, and pyrimidine biosynthesis, KDO *Lipid* A syn: (Kdo)2-lipid A biosynthesis, Pyridoxal sal: pyridoxal 5′-phosphate biosynthesis and salvage, NAD sal III: NAD salvage pathway III (to nicotinamide riboside), ppGpp: ppGpp metabolism, LPS syn: lipopolysaccharide biosynthesis, Mycolate syn: mycolate biosynthesis, Oleate syn IV: oleate biosynthesis IV (anaerobic), (5Z)-Dode syn I: (5Z)-dodecenoate biosynthesis I. Plotting: Cladograms and histograms of LEfSe results were plotted in Galaxy (web application, https://huttenhower.sph.harvard.edu/galaxy/) and taxa names cleaned with INKSCAPE (v0.92.3-1, https://inkscape.org/). PCoAs were plotted using Vega editor (v5.22.1, https://vega.github.io/editor/#/).

**Figure 6:**
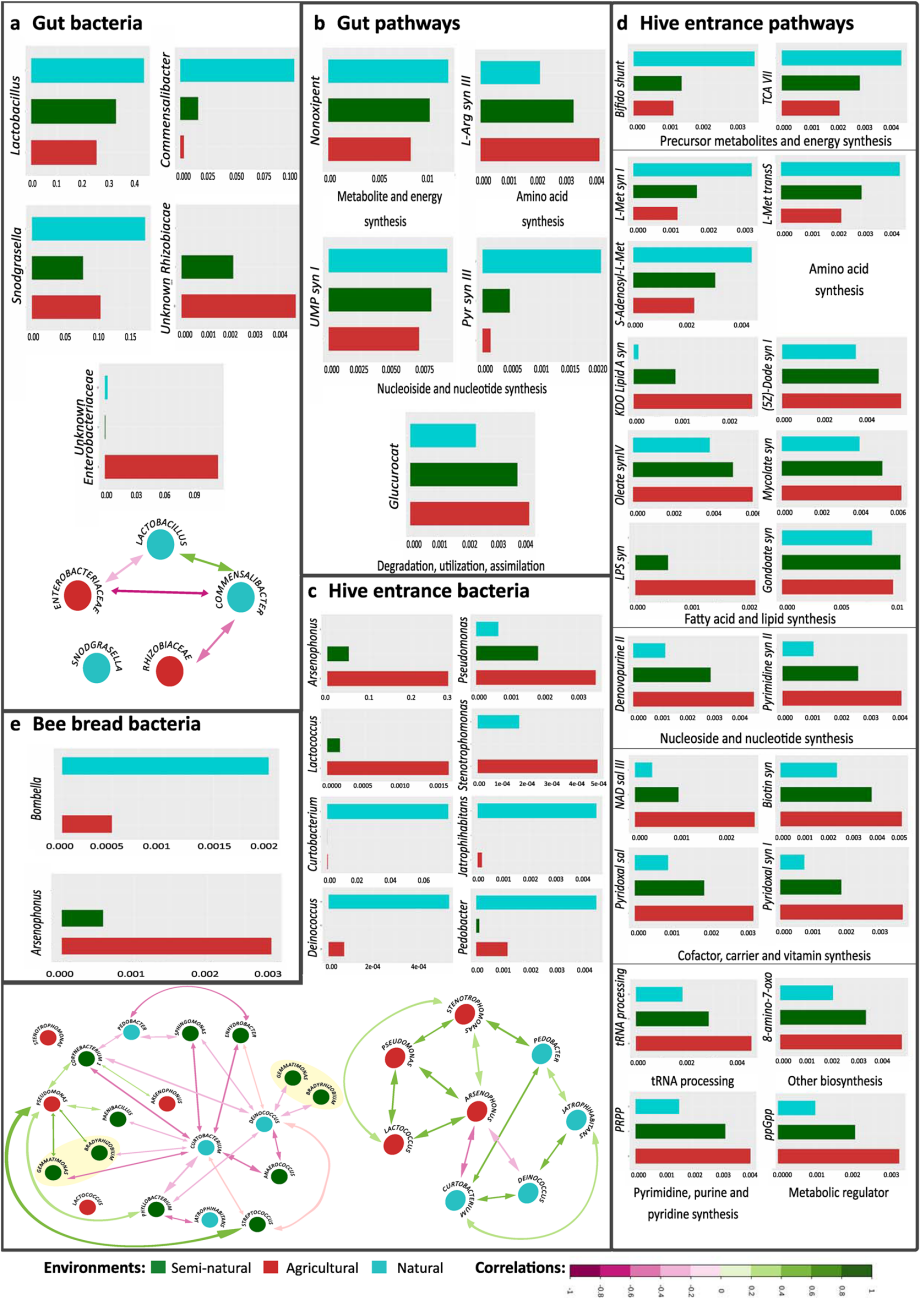
Median relative abundances of bacterial and functional biomarkers showing intermediate values in the semi-natural location for gut, hive entrance and bee bread samples. The scales of the axes vary according to the relative abundance of the plotted feature. Biomarkers were defined as features with relative abundances significantly different across environments, according to LEfSe analysis (Kruskal–Wallis test *p* < 0.05, *LDA* > 3.0). (**a**) Bacterial biomarkers of guts, plus schematic of Spearman correlations (*p* < 0.05). (**b**) Predicted functional biomarkers in the gut. (**c**) Bacterial biomarkers of hive entrances, plus schematic of Spearman correlations (*p* < 0.05) for all environments. (**d**) Predicted functional biomarkers in the hive entrance. (**e**) Bacterial biomarkers in bee bread. Nonoxipent: Pentose phosphate pathway I (non-oxidative branch), L-Arg syn III: l-arginine biosynthesis III (via N-acetyl-l-citrulline), UMP syn I: UMP biosynthesis I, Pyr syn III: Pyrimidine deoxyribonucleotides de novo biosynthesis III, Glucurocat: β-d-glucuronosides degradation. (**d**) Bifido shunt: Bifidobacterium shunt (hexose catabolism), TCA VII: TCA cycle VII (acetate-producers), L-Met syn I: l-methionine biosynthesis I, L-Met transS: l-methionine biosynthesis (transsulfuration), S-Adenosyl-l-Met: S-adenosyl-l-methionine biosynthesis, KDO Lipid A syn: (Kdo)2-lipid A biosynthesis, (5Z)-Dode syn I: (5Z)-dodecenoate biosynthesis I, Oleate syn IV: oleate biosynthesis IV (anaerobic), Mycolate syn: mycolate biosynthesis, LPS syn: lipopolysaccharide biosynthesis, Denovopurine II: superpathway of purine nucleotides de novo biosynthesis II, Pyrimidine syn II: pyrimidine deoxyribonucleotides de novo biosynthesis II, NAD sal III: NAD salvage pathway III (to nicotinamide riboside), Biotin syn: biotin biosynthesis I, Pyridoxal sal: pyridoxal 5′-phosphate biosynthesis and salvage, Pyridoxal syn I: pyridoxal 5'-phosphate biosynthesis I, tRNA processing: tRNA processing, 8-amino-7-oxo: 8-amino-7-oxononanoate biosynthesis I, PRPP: histidine, purine, and pyrimidine biosynthesis, ppGpp: ppGpp metabolism, gondoate syn: gondoate biosynthesis (anaerobic). Plotting: Schematics were done in INKSCAPE (v0.92.3-1, https://inkscape.org/) considering the results of Hmisc and corrplot packages.

Environmental effects were clear, with only *Sphingomonas, Bradyrhizobium* and *Methylobacterium* abundantly present in all environments. Agricultural and semi-natural apiaries were overrun by Proteobacteria and more enriched than natural samples for Firmicutes (*Lactobacillus, Staphylococcus, Streptococcus, Paenibacillus*). The non-natural apiaries differed in bacterial abundances, rather than presence/absence of bacteria. Gammaproteobacteria (mainly *Arsenophonus, Stenotrophomonas* and *Pseudomonas*) were representative of agricultural samples, as was *Lactococcus* (Firmicutes) (Fig. 5a). Natural samples had a divergent microbial profile, with abundance of both Actinobacteria and Bacteroidia classes. The *Aureimonas* and *Deinococcus* genera were significantly enriched in these natural colonies (*LDA* > 3.5) (Fig. 5a) while *Massilia* was slightly enriched (Supplementary Table S2), *Arsenophonus* was absent and *Sphingomonas*, *Phyllobacterium* and *Bradyrhizobium* had overall lower abundances (Supplementary Table S3). The *Sphingomonas* genus (*LDA* > 5.0), the Bacilli class, several Proteobacteria genera, as well as the phyla Firmicutes, Gemmatimonadetes and Actinobacteria were the most significant for semi-natural samples (Fig. 5a). Two genera significant for agriculture, *Arsenophonus* and *Lactococcus*, showed intermediate abundances in semi-natural samples (Fig. 6c). Species wise, *Paenibacillus larvae* and *Lactobacillus kunkeei* (both Bacilli) plus *Corynebacterium afermentans *sub. sp.* afermentans* (Actinobacteria) were semi-natural representatives (*LDA* > 3.0), with *P. larvae* present in natural samples and practically absent in agricultural hives, while *L. kunkeei* and *C. afermentans* were present in agricultural hives but absent in natural hives (Supplementary Table S4).

Concerning interactions among bacteria, correlations were mostly grouped by taxa. Actinobacteria and Bacteroidia such as *Jatrophihabitans, Curtobacterium* or *Hymenobacter* promoted mutual presence and vice versa, while the presence of Alphaproteobacteria was promoted by different Firmicutes (Spearman correlation, *R* > 0, *p* < 0.05). Exclusion, marked by negative correlation, was detected between several Actinobacteria or Bacteroidia versus genera such as *Arsenophonus, Corynebacterium 1, Micrococcus* and *Gaiella* (Fig. 5b).

In the predictive functional profile, natural hives clustered together (explaining 19.53% of dissimilarities) while agricultural samples scattered across the PCo1 axis (Fig. 5c). Diversity of functional profiles was highest in natural hives and lowest in semi-natural (Shannon’s index, *p* < 0.05) (Supplementary Fig. S5b). None of the significantly recruited functions were exclusive to one environment. The only non-ubiquitous function was lipopolysaccharide (LPS) biosynthesis (absent in natural colonies) (Fig. 6d), although certain pathways were also scarce in the other environments (pyrimidine biosynthesis III in agricultural gut and (Kdo)2-lipid A biosynthesis in natural hive entrance) (Fig. 6b,d). Natural colonies exhibited increased frequency of the *Bifidobacterium* shunt, l-methionine biosynthesis (mostly mediated by transsulfuration occurring from oxaloacetate), and a tricarboxylic acid cycle specific to acetate-producers (TCA cycle VII) (Fig. 5d). Agricultural hives possessed enriched synthesis of nucleotides, cofactors, nicotinamide adenine dinucleotide (NAD), membrane components (Kdo2 lipid A, LPS, mycolate) and fatty acids. The tRNA processing pathway resulting in tRNA activation, and the stringent specific ppGpp metabolism were also significantly expressed (Fig. 5d). Semi-natural samples had significant recruitment of the tryptophan 7-halogenase enzyme (Fig. 5e) and of anaerobic gondoate biosynthesis (Fig. 5d) and intermediate values for other pathways enriched in the more extreme environments (Fig. 6b,d).

#### Bee bread samples

Contrasting environments shared similar microbiomes, with differences primarily found in abundances of scarce taxa. *Sphingomonas* and *Methylobacterium* (Alphaproteobacteria) were overall the most abundant genera, followed by *Acinetobacter* in the natural environment and *Bradyrhizobium* in the other two (Supplementary Table S2). The gut core genus *Bombella* was enriched in natural hives, *Arsenophonus* represented the agricultural environment, and no taxa was augmented in semi-natural samples (LDA > 3.0) (Supplementary Fig. S4c).

#### Internal hive air

Environmental effects were scarce for internal air, and only seen in the enrichment of Enterobacteriaceae (mostly *Arsenophonus*), *Curtobacterium* and *Massilia* in agricultural samples (Supplementary Fig. S6).

### Intermediate functional and bacterial community profiles in semi-natural hives

Beta-Diversity results of gut bacterial communities and predicted functions showed that semi-natural samples clustered between natural and agricultural hives (Figs. 3b, 4c). This effect was also apparent for predicted functions of hive entrance (Figs. 3a, 5c) samples. In concordance, semi-natural hives showed intermediate relative abundances for several taxa and functional pathways, while natural and agricultural environments exhibited either lowest or highest relative abundances (Fig. 6). This trend was more evident at a functional (Fig. 6b,d) than at the taxonomic level (Fig. 6a,c). Bacterial representatives showing intermediate abundances for gut samples included *Comensalibacter*, which was enriched in natural, scarce in semi-natural and mostly absent in agricultural samples. An unknown Rhizobiaceae genus exhibited the opposite tendency (enrichment in agriculture and absence or nearly absence in natural samples) (Fig. 6a). Same pattern was observed for *Lactococcus* in hive entrance (Fig. 6b) and *Arsenophonus* in hive entrance (Fig. 6b), bee bread (Fig. 6e) and gut samples (Supplementary Table S2). A less pronounced transition was detected for *Lactobacillus* in gut samples and *Pseudomonas* in the hive entrance. Both were present in all environments but augmented in natural and agricultural environments, respectively, while the semi-natural environment had intermediate abundances. Bee bread and gut samples also showed overall intermediate abundances for some non-environmentally relevant bacteria, such as *Bradyrhizobium* in bee bread and *Gilliamella* in gut (Supplementary Table S2). Besides the aforementioned bacteria showing intermediate abundances, all significantly recruited functions in gut and hive entrance samples had highest or lowest recruitment in natural and agricultural colonies, and intermediate values in the semi-natural environment (Fig. 6b,d). This behaviour was clear in the agricultural recruitment of NAD and Kdo2-lipid A synthesis, both displaying relative mean abundances under 0.001% in natural colonies (Fig. 6d). The exceptions to the rule were the gondoate anaerobic synthesis enriched in semi-natural hive entrance and the *Bifidobacterium* shunt with equally low relative abundances in both anthropized locations (Fig. 6d).

## Discussion

Several studies have revealed anthropization-induced bacterial shifts in the honey bee gut microbiota^14,16,17,19,30^, disturbing gut microbial abundances, composition and functions. In this study, environmental anthropization resulted in the enrichment of potential pathogens and bacteria capable of surviving in contaminated landscapes, as well as recruitment of stress response-related functional pathways in bee gut and hive entrance samples. Importantly, semi-natural hives, despite being genetically identical to the agricultural hives (formed from same origin bees, queens and food reserves) but unrelated to the natural ones, showed intermediate values at taxonomic and functional levels. This mixing of agricultural and natural traits likely stemmed from environmental factors (e.g. pollen diversity or pollution).

Overall, bacterial communities associated with natural hives did not differ from the expected core profile for gut samples^13^, and were devoted to performing essential functions. Bacteria adapted to the natural environment were mainly found in the hive entrance. These natural hives were heavier but less populated than the non-natural beehives. Reduced colony sizes have been associated with *Varroa*-surviving colonies^31^.

Worker guts from the natural environment displayed bacterial profiles associated with good health, and were enriched in Acetobacteraceae and the gut core members *Snodgrasella*, *Lactobacillus* and *Commensalibacter* (involved in nutrient acquisition and immune responses). Enrichment of Acetobacteraceae and Lactobacillaceae has been associated with apiaries found > 1 km away from crops^14^, while *Commensalibacter* enrichment has been related to natural environmental conditions^30^, winter honey bees^32^, and resilience against *European foulbrood*^28^. Some *Lactobacilli* are core members of the honey bee gut microbiome, ferment bee-diet byproducts^9–11^ and can inhibit the pathogen *Paenibacillus larvae* in the gut of larvae^7^. Interestingly, *Paenibacillus* appeared in the natural apiary at low abundances across all the apibiome, except for beebread where its abundances equalled the ones found in agricultural samples. *P. larvae* was also present in natural hive entrance samples, yet no clinical symptoms of American Foulbrood were seen, suggesting spore inactivation or suppression of the pathogen by the bee gut bacterial community. *Lactobacillus* impoverishment (as detected in our agricultural hives) has been detected following antibiotic application^33^. *Snodgrasella* further contributes to improved honey bee gut equilibrium by facilitating biofilm formation in the gut^34^, maintaining anaerobiosis for gut symbionts^9^, and expressing immune genes after *Escherichia coli* infection^35^. Protozoan inhibition in natural honey bee gut through a combined enrichment of *Lactobacillus*, *Commensalibacter* and *Snodgrasella* was previously hypothesised as likely^30^, and known to occur in bumblebees^36^. Consistently, *Commensalibater* and *Lactobacillus* were positively correlated in our study and were dominant in the natural environment. All in all, the consortium of genera found in guts of natural colonies maintained gut environment homeostasis (anaerobiosis and biofilms) and possibly hindered infectious or opportunistic colonizations, supporting honey bee welfare by both suppressing pathogen infections or allowing its tolerance (e.g. *P. larvae*), and by favouring the intake of essential molecules and nutrients. Taken together, this natural taxonomic profile represented a balanced honey bee microbiome. In contrast, agricultural gut bacterial communities were enriched in non-core bacteria, similar to the findings of Muñoz-Colmenero et al.^30^, who also stipulated that anthropic factors could lead to microbial shifts and benefit the proliferation of opportunistic bacteria. The agricultural guts were rich in Enterobacteriaceae and Rhizobiaceae, both anthropization-related, with Rhizobiaceae having been linked to sugar syrup feeding^15^ and Enterobacteriaceae to Coumaphos and Chlorothalonil pesticide usage^18^ as well as to anthropization^30^. Several Enterobacteriaceae species are also opportunistic environmental bacteria and their presence in the honey bee gut has been associated with colony health weakening^37^ and illness^38^. Various Enterobacteriaceae within our samples were negatively correlated with *Commensalibacter* and *Bartonella*. Augmentation of *Bartonella* was detected in winter bees^32^ and in newly emerged bees after nutritional stress and *Nosema ceranae* infection^16^. Thus, whilst *Commensalibacter* maintained homeostasis of natural bacterial communities, *Bartonella* might have performed a similar function in semi-natural colonies.

In summary, enrichment of opportunistic bacteria and reduction of beneficial taxa was evident in agricultural colonies, confirming the detrimental impact of environmental anthropization. In between both extremes, semi-natural profiles were shifting towards natural communities despite genetic similarities and geographic proximity to agricultural colonies. The combined reduction of Enterobacteraceae and enrichment of beneficial *Commensalibacter* and *Lactobacillus* (natural > semi-natural > agriculture), supported the reduced anthropization and more balanced state of semi-natural colonies.

Honey bee gut bacteria show a diminished metabolism^39^ specialised in the usage of recalcitrant compounds (sugars, flavonoid glycosides, etc.) derived from the bee diet^11^. In this sense, most gut organisms conduct anaerobic carbohydrate fermentation^11,39^ while a few take part in biofilm formation or cell adhesion, such as *Gilliamella apicola, Snodgrasella alvi, Lactobacillus* and *Bifidobacterium*^12,39^. In concordance, the natural environment displayed an inherent metabolism supporting the synthesis of indispensable metabolites (UMP and pyrimidine deoxyribonucleotides) and the recruitment of ubiquitous anabolic reactions (non-oxidative branch of the pentose phosphate pathway, PPP). These inherent pathways, depleted in agricultural guts, showed intermediated recruitment in semi-natural samples, suggesting a weakened metabolism in both anthropized locations. Pyrimidine synthesis was especially low in agriculture, which could reflect the diminished *Lactobacillus* abundances in that location as *Lactobacillus* synthesise pyrimidine exclusively^11^. In combination with a less active anabolism, agricultural samples exhibited stress-related pathways. They had increased arginine (Arg) biosynthesis, linked to the aftermath of cold stress in the diptera *Bactrocera dorsalis*^40^, and increased β-d-glucuronoside degradation. Glucuronosides are formed in mammals as byproducts of hepatic glucuronidation, enabling detoxification of unwanted and toxic compounds^41^, and are later excreted into the gut. Recruitment of β-d-glucuronoside degradation within bee guts suggests glucuronoside presence following glucuronidation of toxic molecules by honey bees. The presence of xenobiotics might force the gut bacterial microbiome to invest most of its energy into defence mechanisms, thus neglecting pathways needed for the community to thrive (i.e. functions seen in natural guts). Agricultural honey bees endure under these conditions, but are unable to achieve a sound health state.

Aside from the honey bee gut, other niches within the apibiome also showed a strong environmental impact. The hive entrance, being directly in contact with the hive external area, appeared as a valuable indicator of the landscape. The coastal location of natural colonies (in Unije island) promoted enrichment of mostly aquatic and salt-tolerant bacteria. *Bacteroidetes*, for instance, are often enhanced in haloalkaline habitats^42^ such as our natural shoreline (i.e. high salinity and humidity). More relevant to our study was the decreased presence of the contamination-resistant *Sphingomonas*^43^, and of the potential pathogen* Arsenophonus*^37,38^. The bacteria enriched in agricultural and semi-natural landscapes are common in environments associated with plants (e.g. soils and roots). Resistance to environmental contamination has been reported or suspected for some of these bacteria. Gammaproteobacteria, enriched in agricultural colonies, are highly adaptable chemotrophs suggested to resist unfavourable conditions^42^, while the *Sphingomonas* and *Gemmatimonas* genera in semi-natural hives (enriched and present respectively) are key bacteria in cadmium-contaminated and saline-alkaline stressed soils^42^. Increased *Gemmatimonas* abundance has also been linked to Pyraclostrobin fungicide application^44^ and to long-term use of organic and inorganic fertilisers^45^. *Sphingomonas* are often associated with plant microbiomes, capable of degrading several recalcitrant substances and common helpers of fungi and plants during metal-degradation of soils^43,46^. *Sphingomonas* proliferation in leaf microbiomes has been reported after anti-pathogen treatment of plants^47^. A similar *Sphingomonas* enrichment to the one observed here in semi-natural hives was reported for petroleum-contaminated soils^48^. Indeed, semi-natural and agricultural apiaries were situated near traditional oil and natural gas exploitations^49^.

Enrichment of potential pathogens was another common trait of semi-natural and agricultural hives. Agricultural samples were particularly enriched in *Pseudomonas*^50^ and *Arsenophonus*. In bees, *Arsenophonus* has been linked to increased death rates^26^ and weakened colony health^37^, whilst enrichment of one *Arsenophonus* candidate was associated with increased incidence of Colony Collapse Disorder^38^. Interestingly, one study showed that both environment and social interactions play an important role in honey bee *Arsenophonus* acquisition^26^, and *Arsenophonus* was enriched in all agricultural hive niches. *Arsenophonus* could be a biomarker of anthropization, transmitted through social activities. However, the same study^26^ demonstrated that *Arsenophonus* abundances within honey bee guts were very location-dependent, with nearby hives sharing similar abundances. Thus, we cannot discard the influence of apiary in the differences detected for *Arsenophonus* abundances. Semi-natural hives also revealed several potential honey bee pathogens, including the human-affecting *Streptococcus*^51^, *Anaerococcus*^52^ and *Paenibacillus*. The latter genus posed a great risk to honey bees, since *Paenibacillus larvae* is the causative agent of American Foulbrood (AFB)^53^. If transmitted from entrance to bees, *Paenibacillus* could infect the brood and threaten semi-natural colonies*.* Likewise, the *Lactobacillus kunkeei* present in these semi-natural beehives, if transmitted to the brood, could protect semi-natural colonies by increasing brood resistance against both *P. larvae* and *Nosema ceranae*^7,54^. Further studies would be needed to determine *P. larvae* transmission within beehives. On the contrary, the hive entrances of natural hives exhibited an adapted and overall more pathogen-poor bacterial community, with the exception of *P. larvae* (more abundant than in agriculture). Agricultural hive entrances, compared to semi-natural, had less positive reinforcements against pathogens and contamination (i.e. less abundance of *Lactobacillus kunkeei* and bioremediators). Whilst natural hive entrances recruited functions prevalent among balanced metabolisms (e.g. methionine synthesis), agricultural hive entrances had more active stress-related pathways. They recruited Gram negative bacterial pathways for synthesis of outer membrane components (e.g. Kdo2-lipid A, LPS) as well as the stringent response-inducing ppGpp metabolism, which enables bacterial persistence and pathogenicity^55,56^. The semi-natural profiles shifted towards natural abundances.

Hive internal air and bee bread were the least influenced niches in the apibiome. Internal air, formed by floating abiotic and biotic particles found within hives, was expected to act as an indicator of beehive fitness. Indeed, shifts in airborne microbiome composition have been linked to soil, flora and possibly pollution^57^, as well as urbanisation^58^. Consequently, we expected differences between anthropic and natural landscapes, but the internal hive air microbiota turned out to be mostly stable and largely unaffected by environmental factors. This could be because free floating particles within hives, as happens with pollen granules, might stick to bee bodies and reduce the pool of available bacteria that can be sampled from the in-hive air. Anthropization had a meagre effect on bee bread, but differences among environments were consistent with the changes detected in gut and hive entrance. Bee bread sample composition resembled previous studies^8,23,30^, and overall natural hives had slight enrichment of acidic and sugar-tolerant bacteria while agricultural hives were enriched for *Arsenophonus*, previously described in bee bread obtained from multiple habitats^23^*.* Herein, *Arsenophonus* was most likely transmitted from agricultural bees to their food stores or vice versa, as agricultural bee guts possessed slight *Arsenophonus* enrichment. Contamination of the food reserves by potential pathogens such as *Arsenophonus* might negatively impact honey bee health at the agricultural environment, as consumption of said food could result in gut microbiome dysbiosis or affect the whole apibiome.

One of the main findings of this study was the intermediated relative abundances observed in semi-natural hives for some bacteria (e.g. *Comensalibacter*, *Lactobacillus*, *Arsenophonus*) and several predicted pathways (e.g. UMP and pyrimidine synthesis in guts, mycolate and LPS synthesis in hive entrance), which signalled the existence of an “intermediate state” in the semi-natural area. The intermediate state was more widespread for pathway recruitment than for community composition, indicating an early functional response of the beehive bacterial community. Importantly, 16 days of exposure to an anthropization gradient were sufficient to shift the bacterial fraction of the apibiome of hives. Agricultural hives stayed anthropized while semi-natural bacterial apibiomes became more balanced, resembling the profiles found in a non-anthropized apiary. As a parallel, quick adaptability of the gut microbiome when under pressure has been reported in humans^59,60^ and other mammals^61^. Similarly, honey bee colony-wide analysis of molecular biomarkers also demonstrated an overall increase in the expression of vitellogenin (regulatory protein within bees), antioxidant enzymes and immune proteins in hives situated near areas under wildlife recuperation for the US Conservation Reserve Program^62^. Our results show that placing beehives in less anthropized areas (more natural with less agricultural pressure) would lead to recruitment of beneficial bacteria (e.g. *Lactobacillus* and *Commensalibacter* in honeybee guts) and induce functional reorganisation. Indeed, habitat restoration in agricultural areas by planting native flora can favour recovery of pollinator populations^3^, including wild bees^63^.

## Conclusion

Decreased anthropization of hives increased the relative abundances of beneficial bacteria in all of the sampled hive niches of the apibiome, albeit at different rates, and induced shifts in predicted functional profiles of guts and hive entrances. These results highlight the quick adaptability of honey bee-associated microbiomes. They offer straightforward management strategies to strengthen bee colonies by reducing the impact of anthropization (by planting of indigenous flora around crops, or relocating hives to more natural areas) whilst maintaining current agricultural production. These results also highlight the relevance of the hive as the unit of study for microbial research, as opposed to bees, in order to understand the contribution of each niche to colony health and resilience, as well as the importance of their interactions. Larger longitudinal and long-term analyses considering seasonal changes would enable the identification of global patterns of anthropization and of core microbes within hive niches, and contribute towards the identification of: (1) beneficial profiles that could be targeted to strengthen honey bee health at any time-period, (2) bacteria that weaken colonies, and (3) biomarkers, as *Arsenophonus* appears in this work, indicating the risk status of hives under anthropization. Thus, a checklist of safety and hazard markers could be developed as a management tool to employ as a bioindicator of beehive health.

## Methods

### Hive setup and characterization of study sites

Samples were obtained from 3 apiaries within Croatia. On 20 May 2017, 33 hives were formed in the agricultural region of Marijančaci (45.618139, 18.342667) using 4 capped brood frames with attendant bees, 2 frames of pollen/honey and mated queens. Hives contained one super each. All frames were standard Langstroth, and sister queens and same-origin worker bees were utilised to avoid genetic variation. Hives were moved the next day. Twenty-two hives were relocated 24 aerial km away to the agricultural region of Kozarac (45.717775, 18.680885), henceforth considered the anthropic or agricultural apiary. The remaining 11 colonies were moved to Vardarac (45.621335, 18.775068), which is adjacent to the Kopački Rit Nature Park and 10 aerial km away from Kozarac. These 11 colonies were designated as the semi-natural apiary. Ten additional hives were located in Unije (44.637413, 14.250092), a sparsely populated island in the Adriatic Sea, hereafter referred to as the natural environment. Seven hives were already established on this natural location beforehand. All 7 had (1) lacked management since 2012 (including pesticide treatments), (2) survived *Varroa destructor* infestations and (3) been previously studied by Muñoz-Colmenero et al.^30^. Three additional hives were obtained from the 3 strongest natural hives (n_natural_ = 10). Two supers were added to all 10 hives located in this natural landscape. All three apiaries remained untreated during this experiment.

Apiaries were therefore subjected to an antrophization gradient (agricultural > semi-natural > natural, from most anthropized to least) and surrounded by different flora. Agricultural exploitations and commercial beekeeping practices are regular in Osječko-Baranjska (encompassing both Kozarac and Vardarac apiaries). Grasslands, fruit trees (apple and plum), and intensive commercial crops such as rapeseed, wheat, sunflower, corn, soybeans and barley surrounded the agricultural apiary^30,64,65^. A similar terrain enclosed the semi-natural apiary, although with greater presence of natural flora due to its proximity (< 3 aerial km) to sedges, reed beds, scrublands and wetlands belonging to the Kopački Rit Nature Park^65^. In contrast, the natural location of Unije had pastureland, tufted hair grass, maquis (olive groves), coniferous woodland, and mixed broad-leaved trees (holm oaks)^66^. Arable land was limited to small grassland and shrub plantations around the village^65^. The semi-natural environment was adjacent (< 1 km) to a Special Protection Area (SPA) and Special Areas of Conservation (SAC), while natural hives were situated inside a SPA.

### Measurement of colony strength traits and *Varroa destructor* load

Colony strength parameters were assessed on the 6th of June 2017 following the Liebefeld method^67,69^. Portable electronic scales were used to weigh entire hives. Both sides of all frames were checked for in-field measurement of adult bees, brood and pollen areas (dm^2^), as well as frame walls and bottom board for measurement of adults. Total adult bee, brood and pollen loads were calculated by multiplying the area by 125 (adult bees) or 400 (brood and pollen) as required for standard LR (Langstroth) frames^68^. Varroa load was measured simultaneously by the Powdered Sugar method^69^.

Statistical analyses for colony strength differences among environments were performed in R (Rv3.6.6, 2020-02-29). Differences between environmental means were calculated with post-hoc Tukey’s test for factors meeting ANOVA assumptions, and with Kruskal–Wallis test and post hoc Dunn’s test (using Benjamini–Hochberg correction and rstatix package^70^) for non-normally distributed factors.

### Sample collection and processing for 16S rRNA amplification

Samples were collected in June 6, 2017, 17 days after colony formation. When possible, 4 sample types were collected per hive: young worker bees collected from brood frames (most likely nurses) for gut dissection (G), 8 cm^2^ of bee bread comb randomly selected from a single frame (PB), microorganisms stuck to the hive entrance and collected by swab scrubbing the entire entrance (S), and filters containing vacuum filtered internal air (F) from the hive. Older workers could not be sampled due to the short period elapsed since hive formation. The entrance was swabbed by scrubbing left and right around 6 times per swab tip (3–4 swabs per hive). Sampling of air was done by placing, on top of the honey super, a plastic dome (Lekliško cupola, produced by Dubravko Leskovic) with a perforated side attached to a vacuum hoover (Hf812, J.S.Holdings) with filters. The vacuum was left running for 10 min. Air samples were not collected in the semi-natural environment due to material limitations. Sterilisation of sampling material was undertaken using ethanol 100% and ultraviolet light, and sampling procedures done as established by Muñoz-Colmenero et al.^30^. Samples were frozen in dry ice until their storage at – 20 °C in the Genetics Laboratory of the University of the Basque Country (UPV/EHU). In total, 158 samples were collected from the 43 hives comprising this study.

For each gut sample (N = 43), 10 bee guts were extracted by dissection, pooled in a 1.5 mL tube with 600 µL of 1xPBS, vortexed, and centrifuged at 8000 rpm for 1 min. The supernatant was collected and placed in a clean 1.5 mL tube. This process was repeated once more by adding 400 µl 1xPBS to recover the maximum liquid sample size. The supernatants were combined and stored at − 20 °C. Afterwards, 200 µL of supernatant were taken and used for DNA extraction with a QIAamp^®^ DNA Mini Kit (Qiagen), following the manufacturer’s protocol.

Regarding bee bread (N = 41, missing 2 agricultural samples), 3–4 pollen cells were randomly collected per sample and placed in a 2 mL tube. Cell lysis and DNA extraction was performed following the protocol established by Muñoz-Colmenero et al.^30^.

For hive entrance (N = 42, missing 1 agricultural sample), the cotton parts of two swabs were put in a 2 mL tube, and 400 μL of 1xPBS, 20 μL of Proteinase K and 400 μL of AL Buffer were added. Then the tubes were vortexed and incubated at 56 °C and 900 rpm for 90 min. The resulting supernatant was collected and placed in a clean 2 mL tube. This step was performed twice in order to recover as many microorganisms as possible, after which both supernatants were combined and 400 μL of ethanol were added. The mixture was vortexed and 700 μL from this tube was applied to the QIAamp^®^ DNA Mini Kit (Qiagen) columns to perform the DNA extraction, following the manufacturer’s protocol.

DNA extraction of internal air (N = 32) was performed using a PowerSoil DNA Isolation Kit (PowerSoil). A 2 mL PowerBead Tube with 0.1 mm glass beads (Qiagen) was filled with half a filter and 60 μL of pre-heated (up to 60 °C) lysis buffer (0.1 M Tris–HCl, 0.05 M EDTA, 1.25% SDS, 0.002 mg/ml RNase) and pre-heated solution C1. Samples were homogenised using a Precellys 24 tissue homogenizer (Bertin Technologies) for 4 min. Then the tubes were incubated at 65 °C for 15 min and centrifuged at 10,000×*g* for 30 s. The supernatant was collected and combined with 200 μL of solution C2, after which tubes were again incubated at 4 °C for 5 min and centrifuged at 10,000×*g* for 1 min. Approximately 700 μL of supernatant were then transferred to a 2 ml collection tube, where 1200 μL of pre-shaken solution C4 were added. Extraction was completed according to the MO BIO Laboratories’ protocol (MO BIO Laboratories).

### 16S rRNA gene amplification

Characterization of the bacterial community was performed via amplification of the V4 region of the 16S rRNA gene, using the 515F/806R primer set and protocols described in the “Earth Microbiome Project'' (http://www.earthmicrobiome.org/). These primers contained Illumina sequencing adaptors and a 12 bp barcode sequence bound to the forward primers, allowing sample identification. Amplification was performed with the Illumina Amplicon Protocol, following previously described conditions and using the “with blocking primers'' protocol for bee bread samples^30^. PCR products were examined on a 1.5% agarose gel stained with ethidium bromide. The DNA purification of the PCR products, the preparation of the libraries and the paired-end sequencing were performed at the Sequencing and Genotyping Unit of the University of the Basque Country (SGIKER). Sequencing was performed using an Illumina MiSeq sequencer with a v2 PE 2 × 150 bp kit (300 cycles), and 10% of PhiX was added as the control for the sequencing process.

### Quality checking and processing of 16S sequences

Quality of raw sequences was checked with FastQC High Throughput Sequence QC Report v0.11.5^71^. Demultiplexing of the sequences (without Golay error correction), in-depth sequence quality control by the denoise-paired DADA2 method^72^, and taxonomic assignment were performed in Qiime2 v2.2 (Qiime2-2020.2)^73^ following the recommended thresholds. Amplicon sequence variants (ASVs) present in a single sample were removed. The original feature table was split by sample type to create sample type specific datasets (gut, bee bread, hive entrance and internal air datasets). Phylogenetic trees were generated from these datasets using mafft and fasttree alignment in Qiime2. A Naive Bayes taxonomic classifier was trained with q2-feature-classifier using our sequence data specifications and the 16S rRNA gene reference sequences from the SILVA 132 database clustered at 99% sequence similarity^74^. Taxonomic analysis was preceded by mitochondrial and chloroplast sequence removal. Relative abundances of bacteria were represented for phyla and genera via qiime taxa barplot. The bacterial classes presenting relative abundances of ≥ 0.1% in all sample types were identified and their relative frequencies were visualised in percentages via donut charts using the R packages ggplot2 and dplyr. Some ASVs were only classified up to domain level and grouped as “Bacteria” while the additional group “others” was used to group the remaining ASVs. Genera with mean relative frequencies of ≥ 1.0% in each hive niche were represented via bar plots using ggplot2, while genera with mean relative frequencies of ≥ 0.1% in any of the environments for any of the hive niches were represented via tables.

### Bacterial community diversity and structure

A common sequencing depth for all sample types was determined through alpha rarefaction curves, and utilised to calculate and compare Alpha diversity values among sample types. Samples presenting lower sequence depths were thus filtered out. For each hive niche, bacterial community phylogenetic diversity was determined in Qiime2 via Faith Phylogenetic Diversity (PD)^75^, while Pielou’s evenness index^76^ was used to calculate community evenness. Shannon’s diversity was utilised to account simultaneously for both diversity and evenness. Significant differences were tested based on pairwise Kruskal–Wallis tests and Benjamini & Hochberg False Discovery Rate (BH-FDR) corrected *p-values*. Visualisation was conducted in R with ggplot2 and dplyr. Sequencing depth for each sample type was then determined through alpha rarefaction curves, and rarefied data sets were obtained per sample type for comparison of environments through Alpha and beta Diversity analyses. Samples presenting lower sequence depths were thus filtered out. For each hive niche, Alpha diversity analyses were performed again and for Beta diversity analyses Bray–Curtis distance (community composition dissimilarity) was computed using Qiime2 and visualised as Principal Coordinate Analysis (PCoA) via Vega editor (v5.22.1). Permutational multivariate analysis of variance (PERMANOVA) was calculated in Qiime2 based on rarefied Bray–Curtis matrices and with pairwise BH-FDR correction, to determine whether the bacterial communities between environments differed significantly. Considering that data dispersion can confound PERMANOVA results, homogeneity of group dispersion (PERMDISP)^77^ for environments was calculated with betadisper() on the same matrices. Spearman correlation-based circular UPGMA trees (unweighted pair group method with arithmetic mean) were obtained in Qiime2 and displayed via iTOL (Interactive Tree of Life tool, v6.5.8)^78^. Colors indicating anthropization level within UPGMA trees were added via INKSCAPE (v0.92.3-1).

### Identification of taxa driving environmental differences

The feature frequency tables of each sample type were collapsed at genus level and transformed to relative abundances. Tables were uploaded to the Galaxy web application^79^ where Linear Discrimination Analysis (LDA) size Effect (LEfSe)^80^ was used to identify the bacteria driving the differences among environments. LEfSe uses a non-parametric factorial Kruskal–Wallis sum rank test^81^ to identify differentially abundant taxa, followed by a canonical method to calculate which taxa combinations contribute more to environmental differences. Histograms and cladograms of results were plotted within the Galaxy web application, and taxa names within graphs cleaned using INKSCAPE v0.92.3-1. Taxa were considered significant when nonparametric factorial Kruskal–Wallis test *p-values* ≤ 0.05 and logarithmic *LDA scores* > 3.0. Possible correlations among bacterial biomarkers (taxa presenting *LDA* > 3.0 in LEfSe) in gut and hive entrance samples were studied at genus level by performing the Spearman correlation matrix in R via corr.test() from the Hmisc package, applying BH-FDR correction, and visualized using corrplot package . Non-linear sample distribution was checked before Spearman correlation analysis, using shapiro.test normality test in R^82^ and BH-FDR correction. Mean relative abundances of all significant genera were represented in tables using percentages.

To determine if environmental changes could impact honey bee apibiome functionality, functional prediction of E.C. enzymes^83^ and MetaCyc pathways^84^ were performed for gut and hive entrance samples using the PICRUSt2 v2.3.0-b^85^ qiime2 q2-PICRUSt2 (v2019.10_0) plugin. The resulting E.C. and pathway tables were rarefied for diversity analyses. Functional diversity of environments was determined by Shannon’s diversity index and significance calculated using pairwise Kruskal–Wallis tests. BH-FDR correction was applied to p-values of pairwise analysis. Bray–Curtis distances were visualised via PCoA to determine environmental dissimilarities. Significant predicted function differences among environments were determined by LEfSe, and considered when Kruskal–Wallis *p-value* ≤ 0.05 and *LDA scores* > 3.0. Mean relative abundances of significant features were calculated and visualised as histograms via the ggplot2 and dplyr R packages.

## Supplementary Information

Supplementary Information.

## Data Availability

The 16S rRNA sequences supporting the conclusions of this article were submitted to the Qiita database (https://qiita.ucsd.edu/) with the ID 14084 and are available at the EMBL-EBI with the accession number ERP133378 (https://www.ebi.ac.uk/ena/browser/view/PRJEB48937).
