# Publisher Correction: A short exposure to a semi-natural habitat alleviates the honey bee hive microbial imbalance caused by agricultural stress

**DOI:** 10.1038/s41598-022-25981-x

**Published:** 2022-12-23

**Authors:** June Gorrochategui-Ortega, Marta Muñoz-Colmenero, Marin Kovačić, Janja Filipi, Zlatko Puškadija, Nikola Kezić, Melanie Parejo, Ralph Büchler, Andone Estonba, Iratxe Zarraonaindia

**Affiliations:** 1 Department of Genetics, Physical Anthropology and Animal Physiology, University of the Basque Country (UPV/EHU), Barrio Sarriena s/n, 48940 Leioa, Spain; 2 Instituto de Investigaciones Marinas (CSIC)/Institute of Marine Research, Eduardo Cabello 6, 36208 Vigo, Pontevedra Spain; 3 Faculty of Agrobiotechnical Sciences Osijek, Josip Juraj Strossmayer University of Osijek, V.Preloga 1, 31000 Osijek, Croatia; 4 Department of Ecology, Agronomy and Aquaculture, University of Zadar, Trg Kneza Višeslava 9, 23000 Zadar, Croatia; 5 Department of Fisheries, Apiculture and Special Zoology, Faculty of Agriculture, University of Zagreb, Svetošimunska Cesta 25, 10000 Zagreb, Croatia; 6 Landesbetrieb Landwirtschaft Hessen (LLH), Bieneninstitut, Erlenstraße 9, 35274 Kirchhain, Germany; 7 IKERBASQUE, Basque Foundation for Science, Bilbao, Spain

Correction to: *Scientific Reports* 10.1038/s41598-022-23287-6, published online 06 November 2022

The original version of this Article contained a typographical error.

Figure 5 did not display correctly.

The original Figure 5 and accompanying legend appears below.

**Figure 5:**
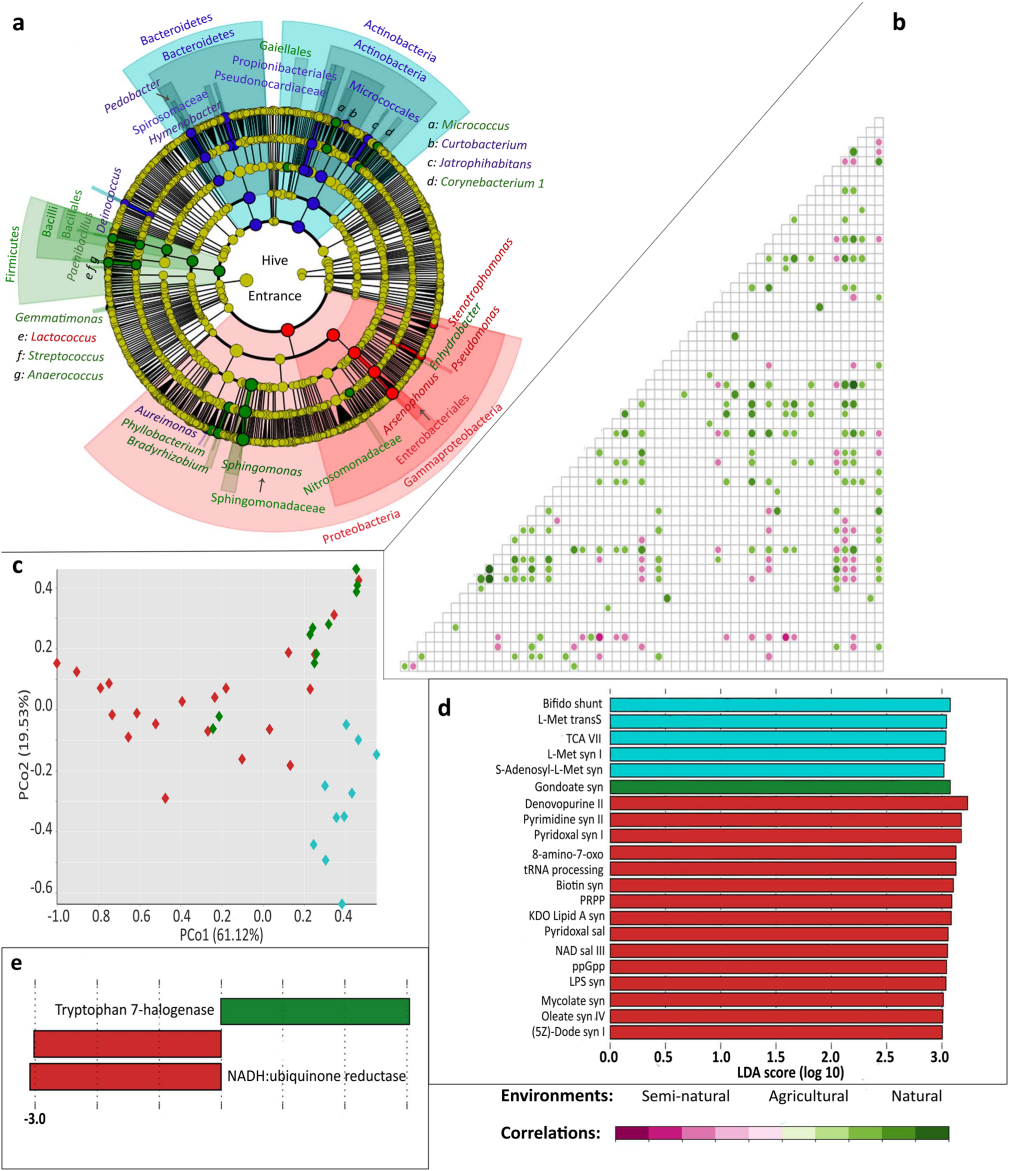
Characterization of the bacterial communities in hive entrance samples. (**a**) Significantly enriched bacteria in each environment, according to LEfSe. Agricultural hives were rich in Gammaproteobacteria and *Lactococcus*. The classes Actinobacteria and Bacteroidia were prevalent in natural samples. Semi-natural samples were enriched in the *Sphingomonas* genus (*LDA* > 5.0), the Bacilli class, genera from the Alphaproteobacteria (*Bradyrhizobium, Phyllobacterium*) and Gammaproteobacteria (*Enhydrobacter*) classes, as well as genera from the Firmicutes, Gemmatimonadetes (*Gemmatimonas* and an uncultured genus), and Actinobacteria phyla. (**b**) Spearman correlation analysis at *p* < 0.05. Positive values were particularly high for *Curtobacterium/Hymenobacter*, *Phyllobacterium/Sphingomonas* and *Phyllobacterium/Bradyrhizobium* interactions (*R* > 0.80, *p* < 0.0001). The most negative interactions were found between *Arsenophonus/Spirosoma* and *Arsenophonus/Nocardioides* (R ⋍ − 0.6, *p* < 0.001). (**c**) Principal Coordinate Analysis (PCoA) of samples according to the predictive functional profile (MetaCyc pathways). (**d**) Significantly recruited functions according to LEfSe. (**e**) Significantly enriched enzymes according to LEfSe. The enzymes Endo X3 (EC 3.1.22.4) and coenzyme Q reductase (EC 7.1.1.2, formerly EC 1.6.5.3) were agricultural representatives, while tryptophan 7-halogenase (EC 1.14.19.9) was enriched in semi-natural samples. Bifido shunt: Bifidobacterium shunt, L-Met transS: l-methionine biosynthesis (transsulfuration), TCA VII: TCA cycle VII (acetate-producers), L-Met syn I: l-methionine biosynthesis I, S-Adenosyl-l-Met: S-adenosyl-l-methionine biosynthesis, Gondoate syn: gondoate biosynthesis (anaerobic), Denovopurine II: purine nucleotides de novo biosynthesis II, Pyrimidine syn II: pyrimidine deoxyribonucleotides de novo biosynthesis II, Pyridoxal syn I: pyridoxal 5′-phosphate biosynthesis I, 8-amino-7-oxo: 8-amino-7-oxononanoate biosynthesis I, tRNA processing: tRNA processing, Biotin syn: biotin biosynthesis I, PRPP: histidine, purine, and pyrimidine biosynthesis, KDO *Lipid* A syn: (Kdo)2-lipid A biosynthesis, Pyridoxal sal: pyridoxal 5′-phosphate biosynthesis and salvage, NAD sal III: NAD salvage pathway III (to nicotinamide riboside), ppGpp: ppGpp metabolism, LPS syn: lipopolysaccharide biosynthesis, Mycolate syn: mycolate biosynthesis, Oleate syn IV: oleate biosynthesis IV (anaerobic), (5Z)-Dode syn I: (5Z)-dodecenoate biosynthesis I. Plotting: Cladograms and histograms of LEfSe results were plotted in Galaxy (web application, https://huttenhower.sph.harvard.edu/galaxy/) and taxa names cleaned with INKSCAPE (v0.92.3-1, https://inkscape.org/). PCoAs were plotted using Vega editor (v5.22.1, https://vega.github.io/editor/#/).

The original Article has been corrected.

