# Supplementary material for: A short exposure to a semi-natural habitat alleviates the honey bee hive microbial imbalance caused by agricultural stress
Source: Sci Rep. 2022 Nov 6;12:18832. doi: 10.1038/s41598-022-23287-6 (PMC9637708; doi:10.1038/s41598-022-23287-6)

Supplementary Information for

A short exposure to a semi-natural habitat alleviates the honey bee hive microbial imbalance caused by agricultural stress

June Gorrochategui-Ortega[1*], Marta Muñoz-Colmenero[1,2], Marin Kovačić[3], Janja Filipi[4], Zlatko Puškadija[3], Nikola Kezić[5], Melanie Parejo[1], Ralph Büchler[6], Andone Estonba[1], Iratxe Zarraonaindia[1,7]

[1]Department of Genetics, Physical Anthropology and Animal Physiology, University of the Basque Country (UPV/EHU), Barrio Sarriena s/n, 48940, Leioa, Spain

[2]Instituto de Investigaciones Marinas (CSIC)/Institute of Marine Research, Eduardo Cabello 6, 36208, Vigo (Pontevedra), Spain

[3]Faculty of Agrobiotechnical Sciences Osijek, Josip Juraj Strossmayer University of Osijek, V.Preloga 1, 31000, Osijek, Croatia

[4]Department of Ecology, Agronomy and Aquaculture, University of Zadar, Trg Kneza Višeslava 9, 23000, Zadar, Croatia

[5]Department of Fisheries, Apiculture and Special Zoology, Faculty of Agriculture, University of Zagreb, Svetošimunska cesta 25, 10000, Zagreb, Croatia

[6]Landesbetrieb Landwirtschaft Hessen (LLH), Bieneninstitut, Erlenstraße 9, 35274, Kirchhain, Germany

[7]IKERBASQUE, Basque Foundation for Science, Bilbao, Spain

[*]Corresponding author.

Email: june.gorrochategui@ehu.eus

Table S1. Beta diversity of the different hive niches by environment using PERMANOVA. Pairwise *p*-values correspond to corrected *p*-values based on the BH-FDR adjustment method. Significance was considered when $p \leq 0.05$: $p \leq 0.05$ (*), $p \leq 0.01$ (**), $p \leq 0.001$ (***). No pairwise comparisons were included for hive air. **Abbreviations:** Env (all by environment), Agri vs Semi (agriculture vs semi-natural), Agri vs Nat (agriculture vs natural), Semi vs Nat (semi-natural vs natural).

	Comparison	PERMANOVA	
		Pseudo-F	***p-value***
**Internal hive air**	Env	1.467	0.111
**Worker gut**	Env	14.545	***
	Agri vs Semi	8.861	***
	Agri vs N	21.238	***
	Semi vs Nat	10.750	***
**Bee bread**	Env	4.777	***
	Agri vs Semi	2.228	0.013**
	Agri vs Nat	8.402	0.0015**
	Semi vs Nat	3.612	0.0015**
**Hive entrance**	Env	11.329	***
	Agri vs Semi	7.626	0.002**
	Agri vs Nat	13.991	0.0015**
	Semi vs Nat	10.444	0.0015**

Table S2. Relative frequencies of genera with ≥ 1% abundance in at least one niche, per environment. The table shows the mean relative frequencies (in %). **Notes: IM** indicates bacteria presenting an intermediate microbiome in semi-natural apiaries, for each sample type (G for gut, H for hive entrance, PB for beebread, F for internal air). **(-)** indicates absence of taxa.

	Internal hive air		Bee bread			IM
Environment	Agricultural	Natural	Agricultural	Semi-natural	Natural	
Acinetobacter	0.677	0.294	0.736	3.467	22.562	*PB
Arsenophonus	1.208	0.008	0.287	0.057	0.000	*PB
Bartonella	0.139	0.074	0.062	0.015	0.030	
Bifidobacterium	0.165	0.025	0.010	0.049	0.005	
Bradyrhizobium	3.056	2.069	3.049	2.764	2.012	*PB
**Uncultured Chitinophagaceae genus**	0.134	33.383	0.104	0.067	0.047	
Commensalibacter	0.067	0.067	0.068	0.007	0.000	
Curtobacterium	0.209	0.000	0.005	0.000	0.008	
**Enterobacteriaceae genus**	0.058	0.057	0.728	0.852	0.813	
Frischella	0.172	0.184	0.146	0.030	0.093	
Gilliamella	1.009	1.273	1.062	0.773	0.438	*PB
Hymenobacter	0.066	0.022	0.023	0.011	0.038	
Lactobacillus	0.436	0.551	0.971	0.927	0.648	*PB
Methylobacterium	15.078	10.440	16.467	14.922	12.393	*PB
Pantoea	0.104	0.000	0.396	0.113	0.437	
Phyllobacterium	2.124	1.363	1.734	1.835	1.582	
Pseudomonas	0.074	0.075	0.964	0.519	1.858	
Snodgrasella	0.590	0.546	0.467	0.295	0.183	*PB
Sphingomonas	63.899	41.693	56.129	52.019	42.905	*PB

	Hive entrance			Gut			IM
**Genus**	**Agricultural**	**Semi-natural**	**Natural**	**Agricultural**	**Semi-natural**	**Natural**	
Acinetobacter	1.073	1.813	1.419	0.023	0.006	0.045	
Arsenophonus	28.605	5.093	0.005	0.021	0.009	0.006	*G
Bartonella	0.282	1.997	0.539	0.473	7.273	0.640	
Bifidobacterium	0.568	0.673	0.279	2.417	2.314	2.813	
Bradyrhizobium	2.014	2.643	0.782	0.178	0.166	0.148	*G
**Uncultured Chitinophagaceae genus**	0.067	0.081	0.694	0.001	0.002	0.004	*G
Commensalibacter	0.101	0.168	0.630	0.331	1.605	10.260	*G
Curtobacterium	0.044	0.005	7.483	0.005	0.000	0.000	
**Enterobacteriaceae genus**	0.201	0.384	0.141	11.287	0.073	0.281	
Frischella	0.156	0.258	0.088	2.910	8.241	3.537	
Gilliamella	1.113	1.479	1.223	37.163	34.288	16.940	*H, *G
Hymenobacter	0.054	0.013	4.949	-	-	-	
Lactobacillus	1.528	1.673	0.601	25.203	32.660	43.451	*G
Methylobacterium	9.769	13.102	8.969	0.834	0.679	0.690	*H
Pantoea	0.099	0.085	0.007	2.452	0.001	0.045	*H
Phyllobacterium	1.402	1.944	0.471	0.092	0.108	0.104	
Pseudomonas	0.357	0.185	0.066	0.020	0.013	0.005	*H, *G
Snodgrasella	0.624	0.891	0.394	10.477	7.815	17.268	
Sphingomonas	37.979	52.569	25.444	3.051	3.218	3.025	

Table S3. Relative frequencies of enriched bacteria genera for all hive niches per environment, expressed in percentages. The table shows the mean relative frequencies (in %) of the bacteria enriched according to LEfSe (Kruskal-Wallis test $p \leq 0.05$ and logarithmic *LDA scores* > 3.0). All hive niches are shown for all enriched bacteria, not only the niches with augmented presence. **Notes: IM** indicates bacteria presenting an intermediate microbiome in semi-natural apiaries, for each sample type (G for gut, H for hive entrance, PB for bee bread, F for internal air). **(-)** indicates absence of taxa.

	Internal hive air		Bee bread			IM
Genus	Agricultural	Natural	Agricultural	Semi-natural	Natural	
Anaerococcus	0.068	0.044	0.002	0.057	0.011	
Arsenophonus	1.208	0.008	0.287	0.057	0.000	*PB
Aureimonas	0.011	0.000	0.000	0.000	0.026	
Bombella	-	-	0.049	0.000	0.205	
Bradyrhizobium	3.056	2.069	3.049	2.764	2.012	*PB
Commensalibacter	0.067	0.067	0.068	0.007	0.000	*PB
Corynebacterium 1	0.178	0.109	0.165	0.130	0.107	*PB
Curtobacterium	0.209	0.000	0.005	0.000	0.008	
Deinococcus	0.000	0.011	0.009	0.027	0.000	
Enhydrobacter	0.170	0.099	0.211	0.229	0.106	
**Enterobacteriaceae genus**	0.058	0.057	0.728	0.852	0.813	
Gemmatimonas	0.066	0.111	0.183	0.165	0.106	*PB
Hymenobacter	0.066	0.022	0.023	0.011	0.038	
Jatrophihabitans	-	-	0.004	0.020	0.051	*PB
Lactobacillus	0.436	0.551	0.971	0.927	0.648	*PB
Lactococcus	0.007	0.000	0.058	0.032	0.127	
Massilia	0.255	0.016	0.037	0.090	0.012	
Micrococcus	0.151	0.053	0.108	0.126	0.079	
Paenibacillus	0.010	0.003	0.039	0.019	0.037	
Pedobacter	0.103	0.000	0.067	0.008	0.000	*PB
Phyllobacterium	2.124	1.363	1.734	1.835	1.582	
Pseudomonas	0.074	0.075	0.964	0.519	1.858	
**Rhizobiaceae genus**	0.048	0.000	0.034	0.075	0.055	
Snodgrassella	0.590	0.546	0.467	0.295	0.183	*PB
Sphingomonas	63.899	41.693	56.129	52.019	42.905	*PB
Streptococcus	0.353	0.439	0.188	0.321	0.285	
Stenotrophomonas	0.014	0.040	0.031	0.000	0.000	*PB

	Hive entrance			Gut			IM
**Genus**	**Agricultural**	**Semi-natural**	**Natural**	**Agricultural**	**Semi-natural**	**Natural**	
Anaerococcus	0.016	0.039	0.000	0.000	0.002	0.001	
Arsenophonus	28.605	5.093	0.005	0.021	0.009	0.006	*G
Aureimonas	0.042	0.004	0.380	0.001	0.000	0.000	
Bombella	0.018	0.011	0.000	0.003	0.000	0.013	
Bradyrhizobium	2.014	2.643	0.782	0.178	0.166	0.148	*G
Commensalibacter	0.101	0.168	0.630	0.331	1.605	10.260	*G
Corynebacterium 1	0.184	0.319	0.028	0.009	0.007	0.004	*G
Curtobacterium	0.044	0.005	7.483	0.005	0.000	0.000	
Deinococcus	0.007	0.000	0.054	-	-	-	
Enhydrobacter	0.048	0.109	0.012	0.004	0.002	0.001	*G
**Enterobacteriaceae genus**	0.201	0.384	0.141	11.287	0.073	0.281	
Gemmatimonas	0.081	0.163	0.020	0.001	0.003	0.000	
Hymenobacter	0.054	0.013	4.949	-	-	-	
Jatrophihabitans	0.017	0.000	0.456	0.001	0.000	0.000	
Lactobacillus	1.528	1.673	0.601	25.203	32.660	43.451	*G
Lactococcus	0.159	0.017	0.000	0.003	0.000	0.001	
Massilia	0.057	0.019	1.173	0.001	0.000	0.000	
Micrococcus	0.097	0.241	0.013	0.004	0.005	0.005	*G
Paenibacillus	0.150	0.249	0.069	0.010	0.000	0.000	*G
Pedobacter	0.118	0.012	0.453	0.006	0.000	0.000	*G
Phyllobacterium	1.402	1.944	0.471	0.092	0.108	0.104	
Pseudomonas	0.357	0.185	0.066	0.020	0.013	0.005	*G
**Rhizobiaceae genus**	0.031	0.071	0.402	0.469	0.213	0.000	*G
Snodgrassella	0.624	0.891	0.394	10.477	7.815	17.268	
Sphingomonas	37.979	52.569	25.444	3.051	3.218	3.025	
Streptococcus	0.108	0.424	0.087	0.011	0.001	0.007	
Stenotrophomonas	0.048	0.000	0.017	0.005	0.000	0.000	

Table S4. LDA scores for enriched bacterial species in hive entrance samples. The table shows LDA values (according to LEfSe with Kruskal-Wallis $p \leq 0.05$ and logarithmic *LDA scores* > 3.0) and mean relative abundances (in %) of the bacterial species enriched in hive entrance samples. All identified species were enriched in semi-natural samples. **Note: (-)** indicates absence of taxa.

	**LDA scores**			**Mean relative abundances (%)**		
	**Agricultural**	**Semi-natural**	**Natural**	**Agricultural**	**Semi-natural**	**Natural**
Corynebacterium afermentans	-	3.82842683755	-	0.015	0.057	0.000
Lactobacillus kunkeei	-	3.72910777176	-	0.024	0.046	0.000
Paenibacillus larvae	-	3.70747533963	-	0.000	0.249	0.063

Fig. S1. Combined violin plots and boxplots for alpha diversity values of the apibiome, by hive niche. A common sequencing depth was used for all niches. Comparisons were performed using Kruskal-Wallis (KW) statistical test, with BH-FDR correction for pairwise KW analyses. Significance was considered when $p \leq 0.05$: $p > 0.05$ (ns), $p \leq 0.05$ (*), $p < 0.01$ (**), $p < 0.001$ (***), $p < 0.001$ (****). Graphics below show non-pairwise results. **a)** Faith Phylogenetic Diversity, for bacterial community biodiversity. Lowest was found in the gut and highest in the hive entrance (pairwise KW, $p < 0.001$). Internal air and bee bread samples presented similar intermediate and more homogeneous biodiversity values (pairwise KW, $p > 0.05$). **b)** Shannon's diversity index. Gut and internal air samples presented the lowest index (pairwise KW, $p > 0.05$) and hive entrance the highest. **c)** Pielou's evenness index. Internal air presented the lowest and gut the highest among all hive niches (pairwise KW, $p > 0.05$).

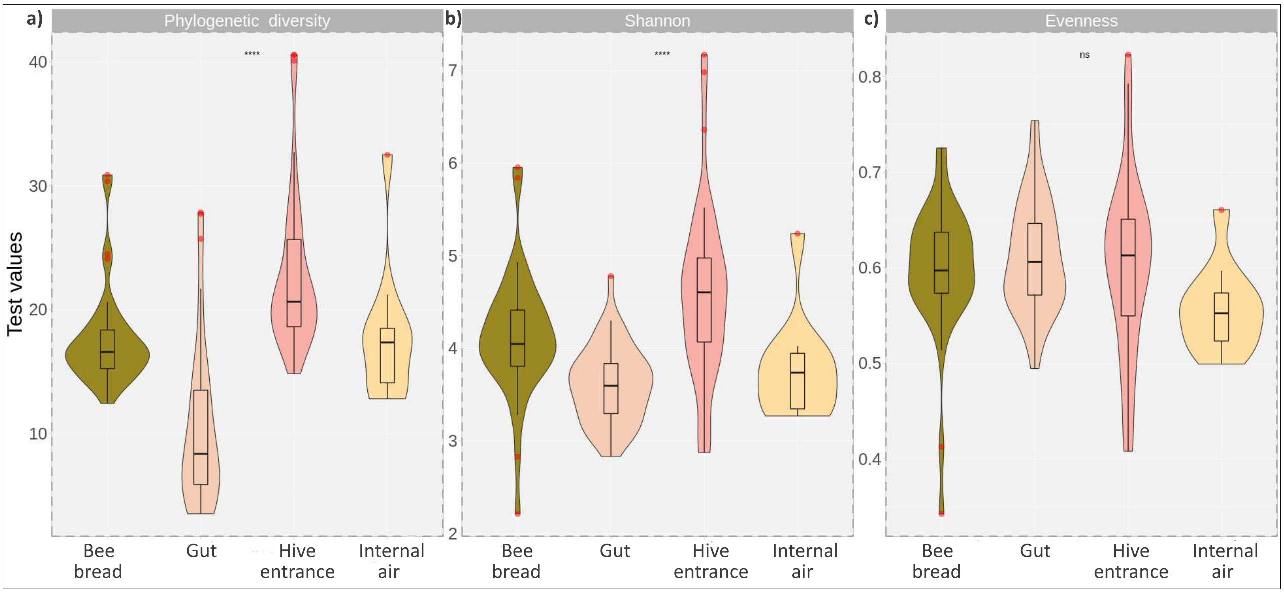

Fig. S2. Bacterial communities of the studied hive niches at genus levels, per sample. A = agricultural apiary, SN = semi-natural apiary, N = natural apiary. **Note:** legend shows the 30 most abundant taxa, starting from the most abundant to the less abundant. The first 8 bacteria have unique colours. After them, colours are repeated in a serial manner. **Plotting:** PCoAs were plotted using Qiime2, and niche and environment names added via INKSCAPE (v0.92.3-1, https://inkscape.org/).

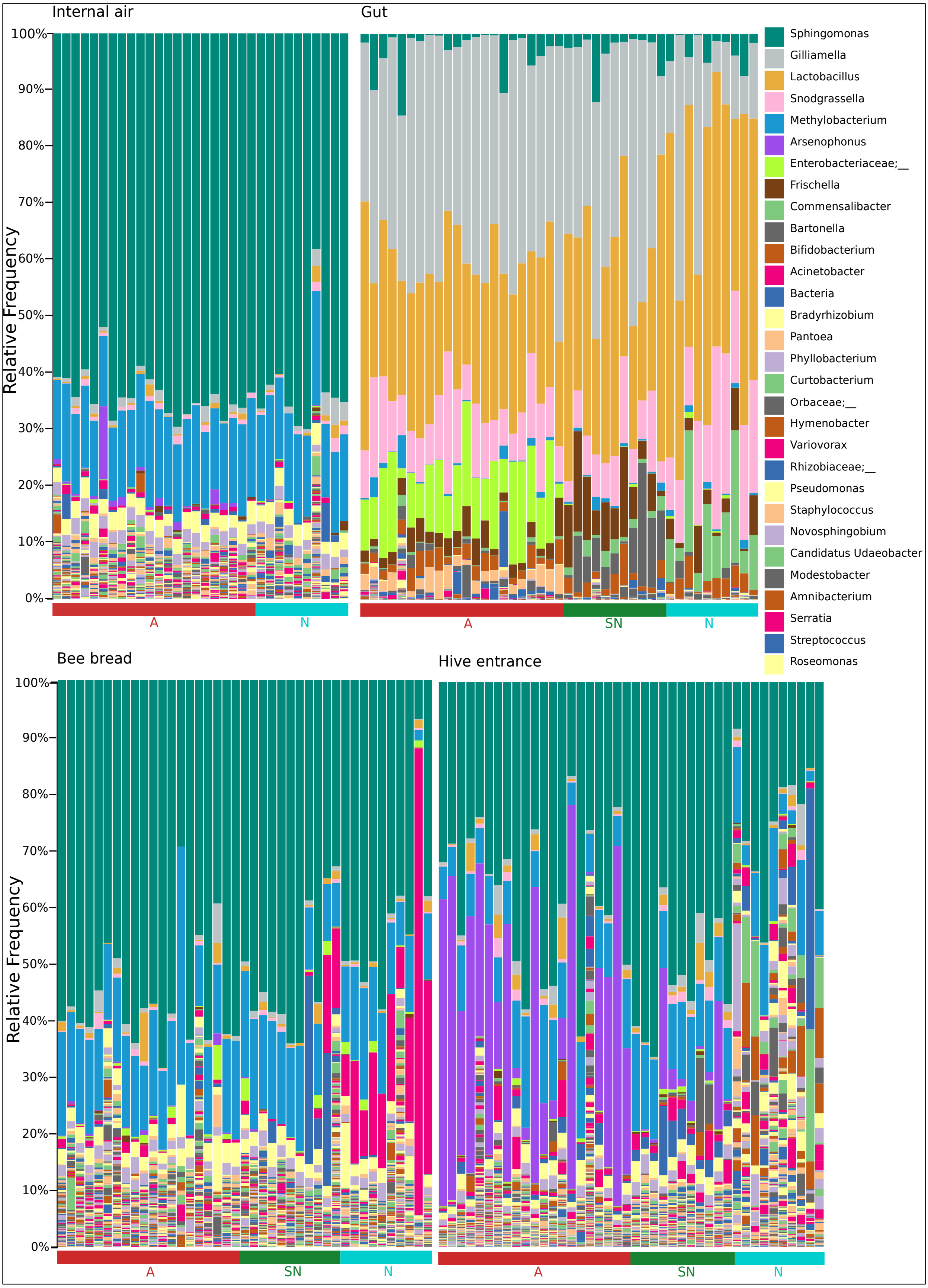
Internal air
Gut
Bee bread
Hive entrance
Relative Frequency
100%
90%
80%
70%
60%
50%
40%
30%
20%
10%
0%
A
SN
N
Sphingomonas
Gilliamella
Lactobacillus
Snodgrassella
Methylobacterium
Arsenophonus
Enterobacteriaceae;__
Frischella
Commensalibacter
Bartonella
Bifidobacterium
Acinetobacter
Bacteria
Bradyrhizobium
Pantoea
Phyllobacterium
Curtobacterium
Orbaceae;__
Hymenobacter
Variovorax
Rhizobiaceae;__
Pseudomonas
Staphylococcus
Novosphingobium
Candidatus Udaeobacter
Modestobacter
Amnibacterium
Serratia
Streptococcus
Roseomonas

Figure S3. Violin plots for alpha diversity values in each hive niche, by environment. Comparisons were performed using Kruskal-Wallis statistical test, with corrected p-values based on the BH-FDR for pairwise analysis. Significance was considered when $p \leq 0.05$: $p > 0.05$ (ns), $p \leq 0.05$ (*), $p < 0.01$ (**), $p < 0.001$ (***), $p < 0.001$ (****). Graphics below show pairwise results on top, while general Kruskal-Wallis results are indicated on the bottom right. **a)** Evenness of bacterial communities, as determined by Pielou's evenness index. No significant tendencies were observed in bee bread and internal air samples. **b)** Faith Phylogenetic Diversity, for bacterial community biodiversity. Significant differences were only detected in hive entrance, but agricultural gut samples did seem to present overall slightly higher and more variable Faith PD values. **c)** Shannon’s diversity index, considering both phylogenetic diversity and evenness.

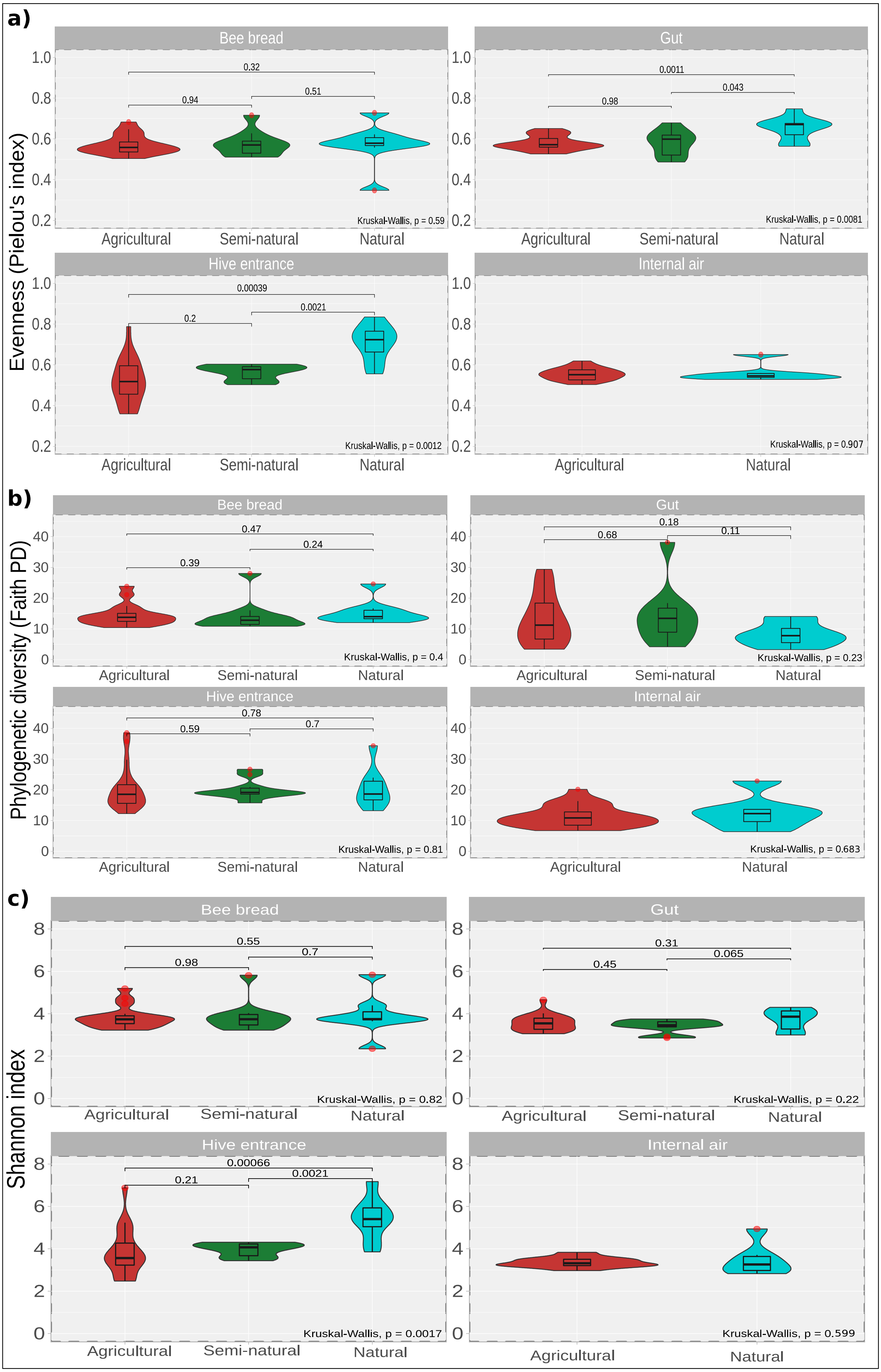

a)
Evenness (Pielou's index)
Bee bread
0.32
0.94
0.51
Kruskal-Wallis, p = 0.59
Gut
0.0011
0.98
0.043
Kruskal-Wallis, p = 0.0081
Hive entrance
0.00039
0.2
0.0021
Kruskal-Wallis, p = 0.0012
Internal air
Kruskal-Wallis, p = 0.907
Agricultural
Semi-natural
Natural
b)
Phylogenetic diversity (Faith PD)
Bee bread
0.47
0.39
0.24
Kruskal-Wallis, p = 0.4
Gut
0.18
0.68
0.11
Kruskal-Wallis, p = 0.23
Hive entrance
0.78
0.59
0.7
Kruskal-Wallis, p = 0.81
Internal air
Kruskal-Wallis, p = 0.683
c)
Shannon index
Bee bread
0.55
0.98
0.7
Kruskal-Wallis, p = 0.82
Gut
0.31
0.45
0.065
Kruskal-Wallis, p = 0.22
Hive entrance
0.00066
0.21
0.0021
Kruskal-Wallis, p = 0.0017
Internal air
Kruskal-Wallis, p = 0.599

Figure S4. Characterization of bacterial communities present in bee bread samples. a) PCoA of Bray-Curtis distances. **b)** UPGMA tree of Bray-Curtis distances. **c)** LEfSe analysis (nonparametric factorial Kruskal-Wallis test $p < 0.05$ and logarithmic LDA > 3.0) only identified two significantly enriched taxa. LEfSe results are plotted according to phylogeny. The bigger the LDA value obtained for a feature, the more significant. Only significant features are plotted in the histogram. **Plotting:** the PCoA was plotted using Vega editor (v5.22.1, https://vega.github.io/editor/#/). UPGMA tree was plotted in iTOL (v6.5.8, https://itol.embl.de/) and internal colors added via INKSCAPE (v0.92.3-1, https://inkscape.org/). Histogram of LEfSe results was plotted in Galaxy (web application, https://huttenhower.sph.harvard.edu/galaxy/) and taxa names cleaned with INKSCAPE.

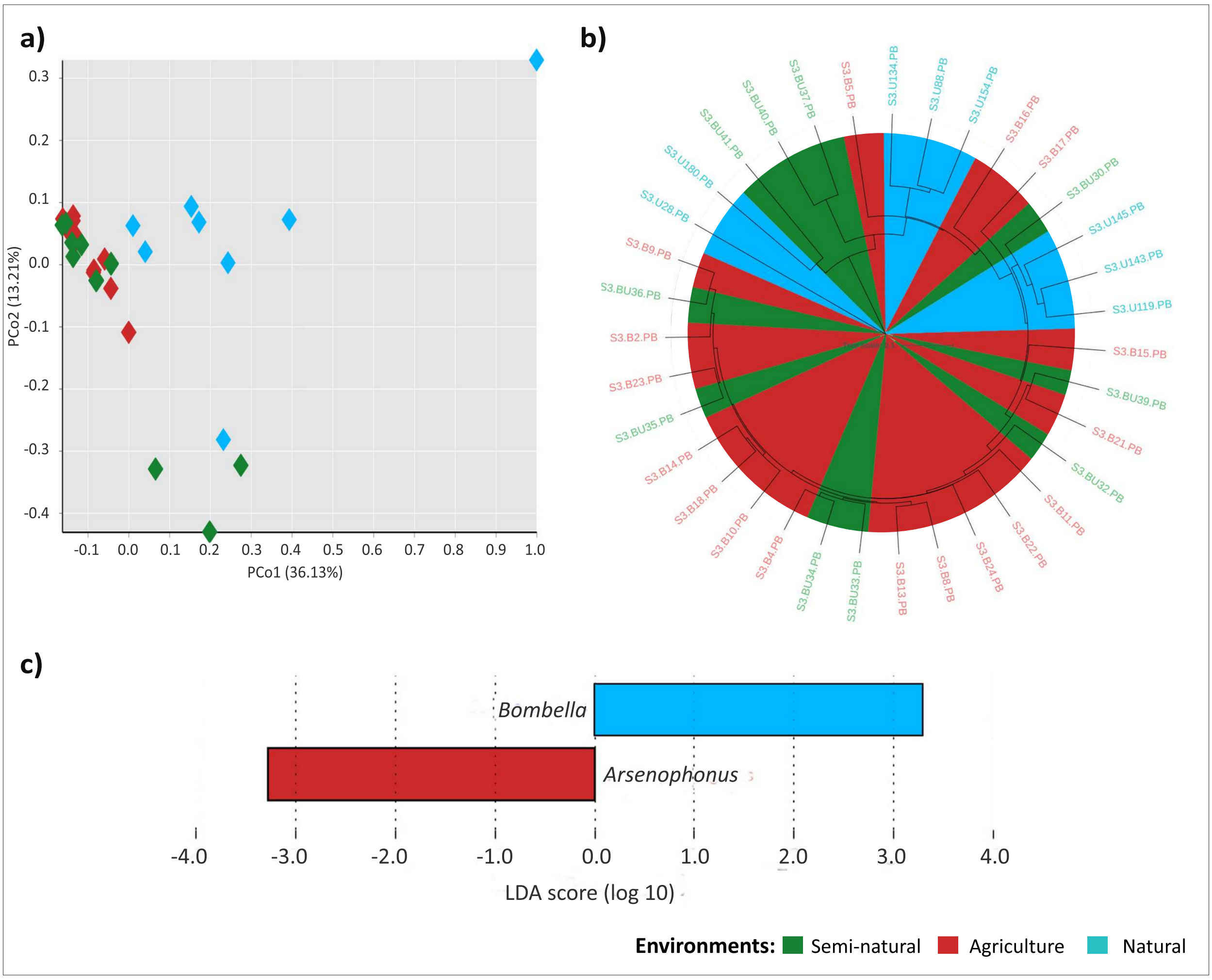

Figure S5. Combined violin plots and box-plots for Shannon's index values (alpha diversity) of the predicted functional pathways, per environment. Comparisons were performed using Kruskal-Wallis statistical test, with corrected p-values based on the BH-FDR for pairwise analysis. Significance was considered when $p \leq 0.05$: $p > 0.05$ (ns), $p \leq 0.05$ (*), $p < 0.01$ (**), $p < 0.001$ (***), $p < 0.001$ (****). Graphics below show non-pairwise results on top, while general Kruskal-Wallis results are indicated on the bottom right. **a)** Gut samples. **b)** Hive entrance samples.

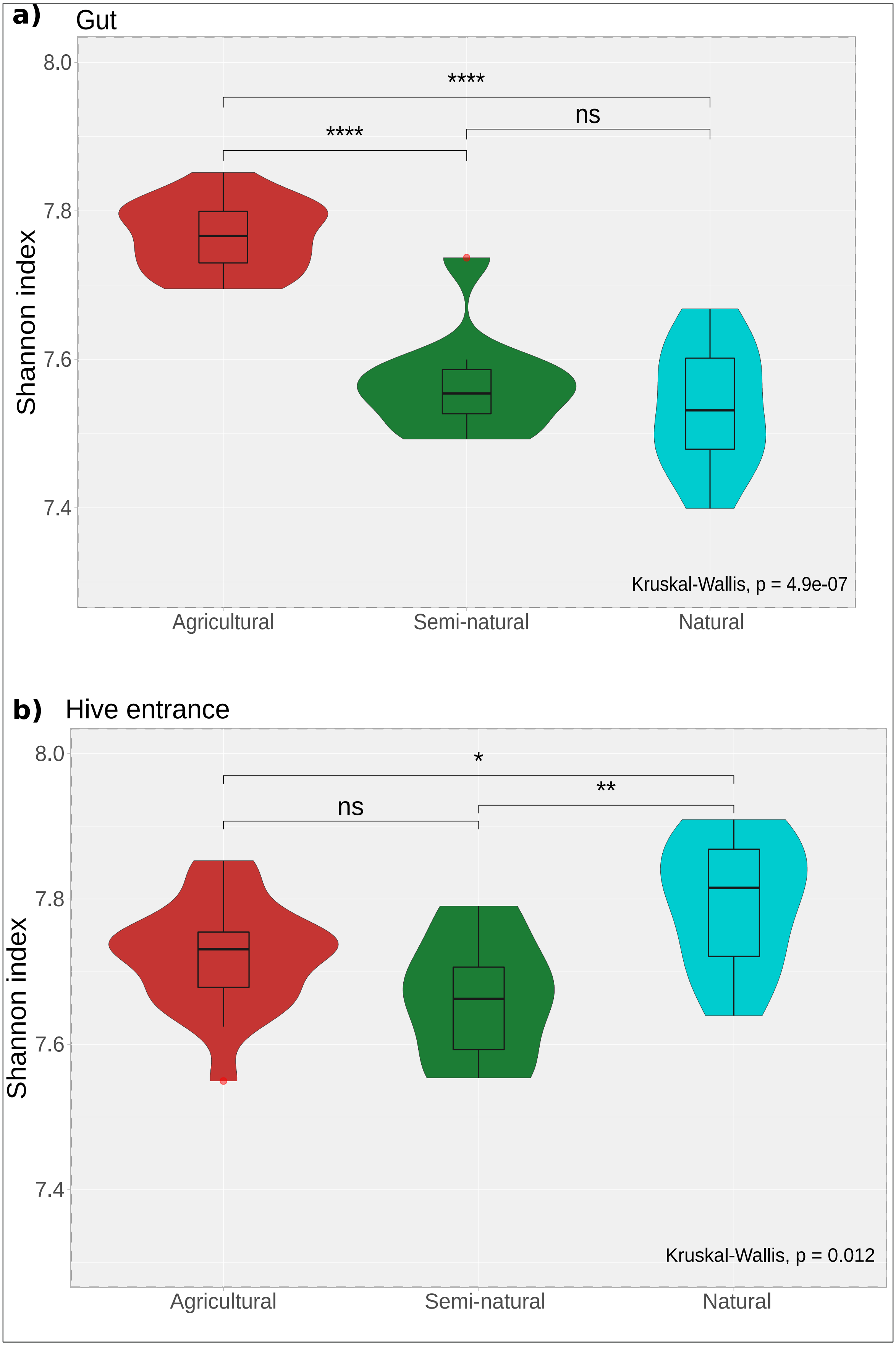

a)
Gut
Shannon index
8.0
7.8
7.6
7.4
****
ns
****
Kruskal-Wallis, p = 4.9e-07
Agricultural
Semi-natural
Natural
b)
Hive entrance
Shannon index
8.0
7.8
7.6
7.4
*
**
ns
Kruskal-Wallis, p = 0.012
Agricultural
Semi-natural
Natural

Figure S6. Characterization of agricultural and natural internal hive air samples, for relative bacterial abundances. No semi-natural hives could be sampled. **a)** PCoA of Bray-Curtis distances. **b)** UPGMA tree of Bray-Curtis distances. **c)** LEfSe analysis (non-parametric factorial Kruskal-Wallis test $p < 0.05$ and logarithmic LDA > 3.0) only presented significant taxa in agricultural colonies. **Plotting:** the PCoA was plotted using Vega editor (v5.22.1, https://vega.github.io/editor/#/). UPGMA tree was plotted in iTOL (v6.5.8, https://itol.embl.de/) and internal colors added via INKSCAPE (v0.92.3-1, https://inkscape.org/). Histogram of LEfSe results was plotted in Galaxy (web application, https://huttenhower.sph.harvard.edu/galaxy/) and taxa names cleaned with INKSCAPE.

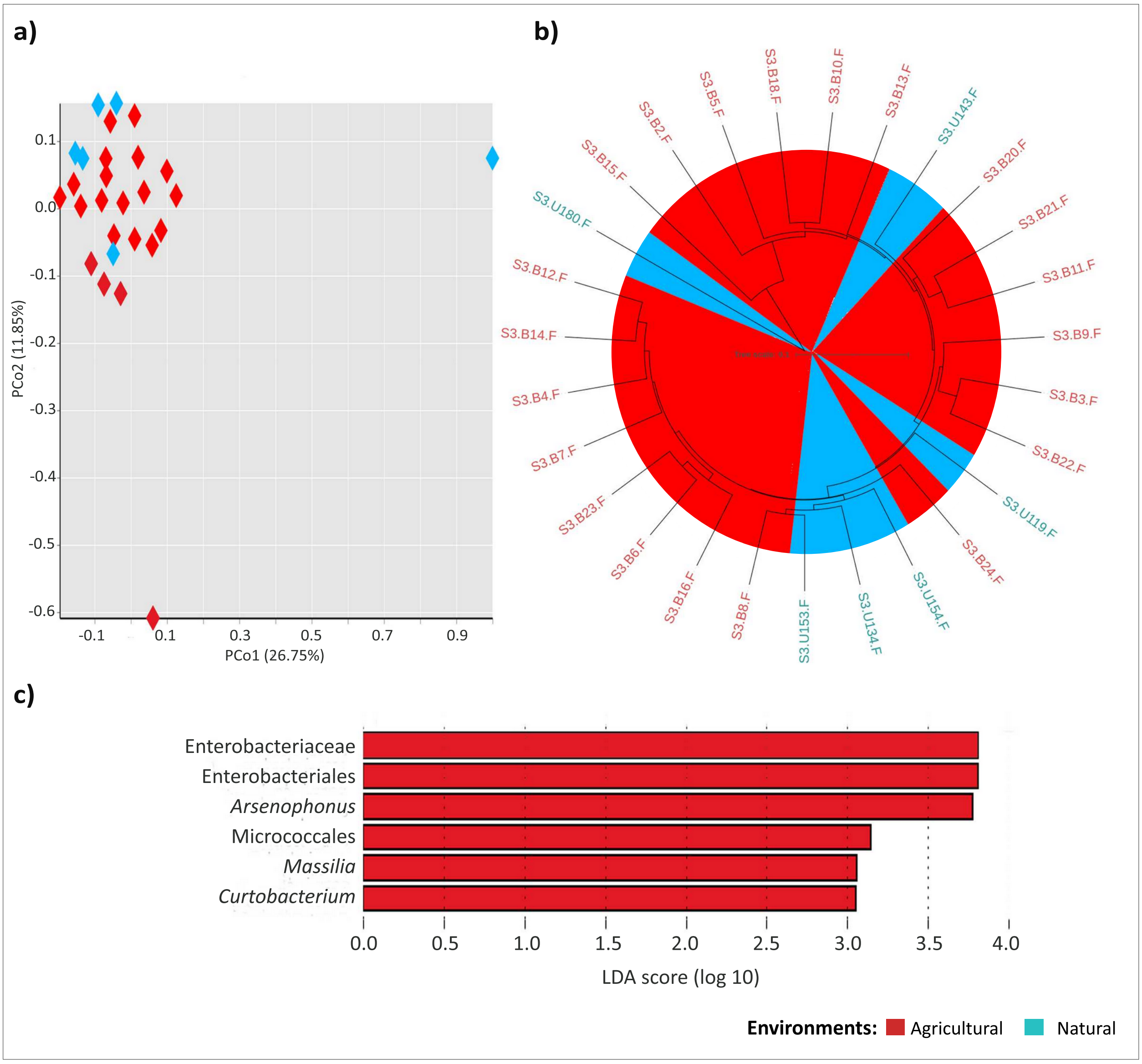

Supplement: Supplementary file 1 — Supplementary Information. [file 41598_2022_23287_MOESM1_ESM.pdf]
